# Coupled Downscaled Climate Models and Ecophysiological Metrics Forecast Habitat Compression for an Endangered Estuarine Fish

**DOI:** 10.1371/journal.pone.0146724

**Published:** 2016-01-21

**Authors:** Larry R. Brown, Lisa M. Komoroske, R. Wayne Wagner, Tara Morgan-King, Jason T. May, Richard E. Connon, Nann A. Fangue

**Affiliations:** 1 California Water Science Center, United States Geological Survey, Sacramento, California, United States of America; 2 National Research Council under Contract to Southwest Fisheries Science Center, National Marine Fisheries Service, National Oceanic and Atmospheric Administration, La Jolla, California, United States of America; 3 Department of Wildlife, Fish, and Conservation Biology, University of California, Davis, California, United States of America; 4 Department of Geological Sciences, University of Texas, Austin, Texas, United States of America; 5 School of Veterinary Medicine, University of California, Davis, California, United States of America; The Australian National University, AUSTRALIA

## Abstract

Climate change is driving rapid changes in environmental conditions and affecting population and species’ persistence across spatial and temporal scales. Integrating climate change assessments into biological resource management, such as conserving endangered species, is a substantial challenge, partly due to a mismatch between global climate forecasts and local or regional conservation planning. Here, we demonstrate how outputs of global climate change models can be downscaled to the watershed scale, and then coupled with ecophysiological metrics to assess climate change effects on organisms of conservation concern. We employed models to estimate future water temperatures (2010–2099) under several climate change scenarios within the large heterogeneous San Francisco Estuary. We then assessed the warming effects on the endangered, endemic Delta Smelt, *Hypomesus transpacificus*, by integrating localized projected water temperatures with thermal sensitivity metrics (tolerance, spawning and maturation windows, and sublethal stress thresholds) across life stages. Lethal temperatures occurred under several scenarios, but sublethal effects resulting from chronic stressful temperatures were more common across the estuary (median >60 days above threshold for >50% locations by the end of the century). Behavioral avoidance of such stressful temperatures would make a large portion of the potential range of Delta Smelt unavailable during the summer and fall. Since Delta Smelt are not likely to migrate to other estuaries, these changes are likely to result in substantial habitat compression. Additionally, the Delta Smelt maturation window was shortened by 18–85 days, revealing cumulative effects of stressful summer and fall temperatures with early initiation of spring spawning that may negatively impact fitness. Our findings highlight the value of integrating sublethal thresholds, life history, and *in situ* thermal heterogeneity into global change impact assessments. As downscaled climate models are becoming widely available, we conclude that similar assessments at management-relevant scales will improve the scientific basis for resource management decisions.

## Introduction

Global climate change is driving rapid shifts in environmental conditions, which cause impacts on organisms across a variety of scales [1–3]. Managers and scientists are tasked with forecasting future environmental conditions, assessing the possible impacts of such conditions on sensitive species and ecosystems, and developing strategies to mitigate those impacts [4]. A critical component of accomplishing these objectives is assessing future habitat suitability given possible scenarios of climate change and a range of reasonable management actions to mitigate impacts. Such choices are especially critical when managers must consider trade-offs between preserving species and ecosystems and other human values (e.g., agriculture, municipal water supply) and logistical constraints (e.g., limited restoration or conservation resources).

Global climate models (GCMs) have offered key insights into possible global changes in temperature, precipitation and other conditions [5–7]. GCMs utilize data and forecast future conditions over broad geographic scales; however, predicted impacts can vary substantially among regions and ecosystems [5]. Managers and scientists frequently need to evaluate impacts and make conservation decisions over regional or local scales, for example, in specific watersheds or ecoregions. Recent advances in methods to obtain local-scale weather and climate from regional-scale atmospheric variables provided by GCMs (“downscaling”) have begun to address this gap and relatively small-scale evaluations are becoming possible [8–11].

Robust climate change predictions for specific areas or ecosystems may be especially important in highly managed systems. In this context, managers may focus on addressing local stressors in efforts to maintain or enhance ecosystem resiliency in the face of other global stressors over which they have less immediate control [12]. Climate change stressors can include multiple abiotic factors (e.g., temperature, salinity, oxygen) that may differentially impact species and ecosystems of interest, such that one may be the most critical focal stressor to understand and, if possible, alleviate. Therefore, effective prioritization of actions necessitates a robust understanding of the projected stressor landscape on scales relevant to management goals.

Understanding climate change effects may be particularly difficult for highly dynamic ecosystems such as estuaries that are driven by a multitude of interacting oceanic, freshwater and terrestrial inputs [13]. These interactions create complex environments with strongly localized conditions that move and change on multiple time scales, ranging from diurnal tides [14] to decadal climate cycles [15,16]. Thus, the effects of climate change on estuaries and other highly dynamic systems can be difficult to assess from large-scale GCMs or even regionally downscaled data. As a result, the possible effects of climate change on estuarine environments have been addressed in a general sense [17,18], but have not been explored in detail for specific estuaries with a few exceptions [9,19]. However, accurate projections are particularly important for estuarine managers because estuaries often have high ecological and societal value, while also being among the most human-impacted habitats globally [20]. Addressing this complexity of estuarine drivers requires development of downscaled climate models in combination with understanding of species ecological requirements. Such linked information can then be used to provide key insights into future conditions under multiple climate change and management scenarios [8,9,21,22].

Clearly, even with accurate predictions of future abiotic conditions, assessing effects on an ecosystem requires knowledge of the responses of individual species to changing conditions. Sensitivity to climate change stressors can vary among and within taxa due to physiology, ecology, life history or other factors [23,24], such that species of conservation concern may exhibit differential responses. For example, physiological tolerances, acclimatization capacity, sublethal stress effects and adaptive potential can be important influences on climate change stressor effects [25–27], but impacts may also be mediated through species-specific phenology or behavior, such as seasonal timing of sensitive life stages [28,29] or abilities to seek better habitat when environmental conditions become sub-optimal [30]. Therefore, detailed knowledge of species’ biology, in conjunction with their capacity to cope with the environmental shifts they are projected to experience *in situ*, can greatly assist scientists and managers in understanding potential biological impacts such as shifts in habitat suitability. Such information can help identify opportunities for mitigation.

**Fig 1:**
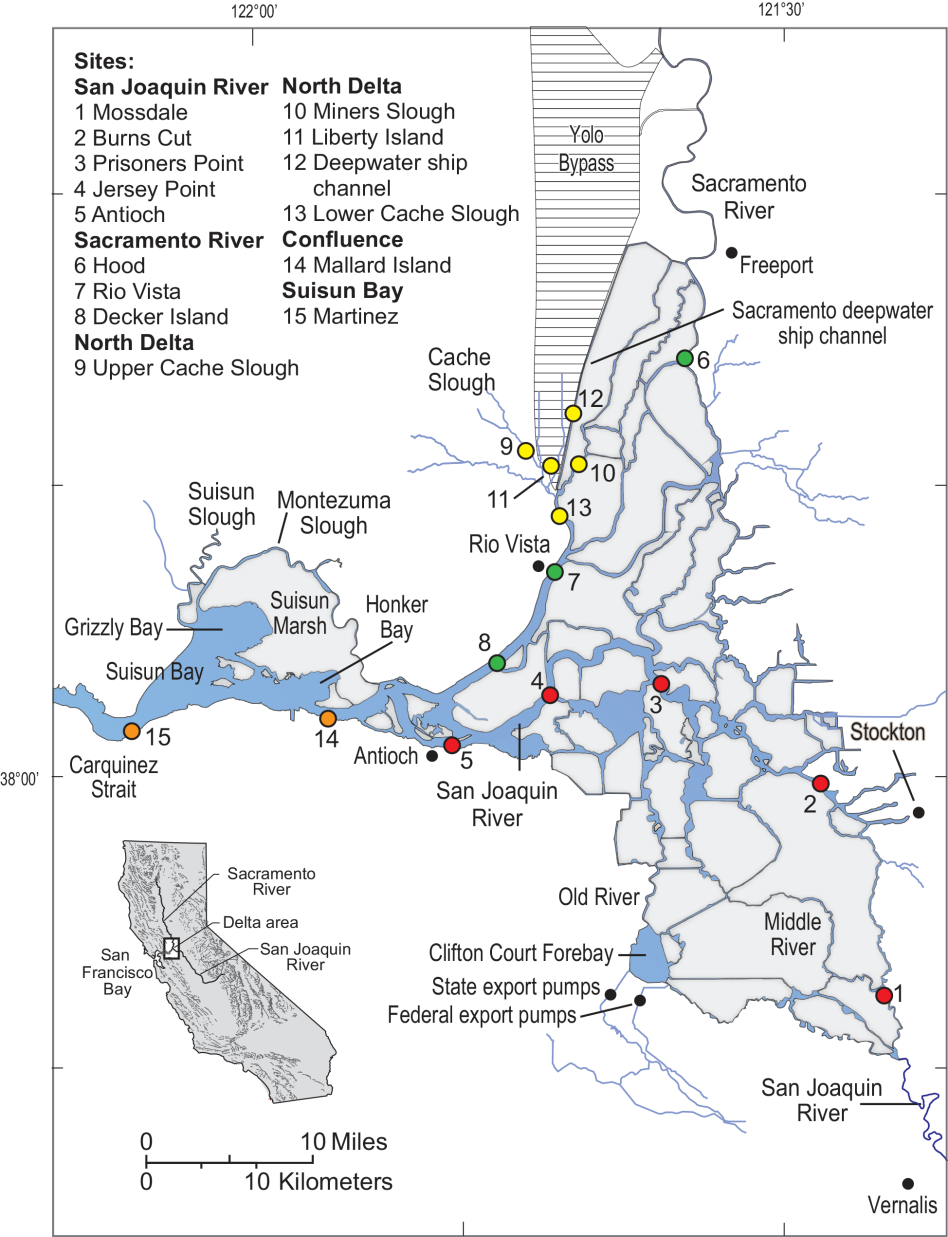
Site map of the Delta showing the locations where water temperature models were developed for the San Joaquin River (red circles), Sacramento River (green circles), North Delta (yellow circles), and the confluence and Suisun Bay (orange circles).

In this study, we utilized regionally downscaled climate change model outputs to forecast water temperatures at fixed locations in the upper San Francisco Estuary (SFE), specifically the Sacramento-San Joaquin Delta and Suisun, Grizzly and Honker Bays (Fig 1; hereafter “Delta”). We relate estimated water temperatures to multiple thermal physiological thresholds (lethal, sublethal, reproductive and development) of Delta Smelt (*Hypomesus transpacificus)* across life stages to assess future thermal habitat suitability within different regions of the upper SFE. We chose Delta Smelt as our study organism because it is an endemic estuary-dependent species of high management concern in California (see below for details). As one of multiple declining native SFE fish species, the status of the Delta Smelt population is considered an ecosystem health indicator, with its recovery strongly tied to regional and statewide conservation plans [31]. In addition to current human stressors, critical aquatic habitat for Delta Smelt and other sensitive native fishes will be subject to altered environmental conditions due to climate change [9,32,33]. In particular, increases in water temperatures may threaten fishes [34] because temperature is a key determinant of fish survival and performance [35,36], resulting in fishes being generally adapted to the water temperatures that they routinely experience [37,38]. Integrating physiological thresholds, natural history, and spatial variability in thermal habitat suitability provides mechanistic insight into global change impacts of this endemic species inhabiting a heterogeneous environment.

## Materials and Methods

### General approach

Our approach was to use outputs of selected global climate change models to drive empirically derived water temperature models for specific locations in the upper SFE where empirical data were available. The modeled water temperature data were then assessed against physiological and behavioral endpoints determined from laboratory- and field-based studies of Delta Smelt. These assessments were based on endpoints relevant to the life history of Delta Smelt and the current distribution of the species in the estuary.

We selected scenarios that bracketed the likely range of future conditions, specifically with regard to air temperature and precipitation changes. A general tendency of GCM projections over northern California is towards little precipitation change from models with smaller warming trends and less precipitation from models with greater warming [39,40]. By selecting two models from near the two extremes of this tendency and two different emission scenarios, we cover a wide range of possible conditions to reflect the uncertainties in GCMs and emissions of greenhouse gases [9].

### Climate change scenarios

Projected scenarios of daily air temperatures were derived from simulations of 21st Century climate by two GCMs under each of two future global greenhouse-gas emissions scenarios from the IPCC’s Fourth Assessment Report [6]. The GCMs used were the Geophysical Fluid Dynamics Laboratory (GFDL) CM2.1 coupled ocean-atmosphere GCM [41] and the National Center for Atmospheric Research Parallel Climate Model (PCM) coupled ocean-atmosphere GCM [42]. The GFDL model is considered strongly sensitive to greenhouse-gas emissions among those considered by the IPCC [6] and produces larger changes in climate compared to other models with the same change in greenhouse-gas emissions. The PCM model is considered to have low sensitivity to greenhouse-gas emissions, producing smaller changes in climate than other models for the same change in greenhouse-gas emissions.

We considered two scenarios of greenhouse-gas emissions available from the IPCC [43]. Climate data from simulations by these two models, under A2 (continually increasing) and B1 (leveling by mid-century) greenhouse-gas emissions scenarios, were obtained from the Program for Climate Diagnosis and Intercomparison at the Lawrence Livermore National Laboratory [44]. These two scenarios largely bracket the range of recent climate-change projections for California, with the GFDL A2 scenario being near the warmer and drier end of current projections and the PCM B1 scenario being near the less-warm and less-dry end of projections [45]. These scenarios are also included in several recent assessments of climate change for California [45,46], which allows for integration of our results with climate change planning for the state. The A2 scenario assumes a very heterogeneous world economy with high population growth and resulting greenhouse-gas emissions that accelerate through the remainder of the century, representing a reasonable estimation of a worst-case scenario. Subsequently, Raupauch et al. [47] showed that during the past decade, emissions have actually exceeded those represented by A2, so our results may underestimate the most extreme possible effects on Delta Smelt. The most recent IPCC assessment [7] was issued after our scenario calculations were completed and indicate that the GFDL A2 scenario can now be considered intermediate-high; thus, we consider the results of our analysis conservative with respect to the effects of climate change in the upper SFE [45]. The B1 scenario assumes a more resource efficient future with lower population growth resulting in emissions leveling off by the end of the century.

### Downscaling

The GCM outputs were “downscaled” by the method of Constructed Analogues [48,49]. Downscaling refers to the transformation of simulated climate variables from the spatial scale of GCMs to estimates of climate at smaller spatial scales. The Constructed Analogues approach yields particularly realistic temperature and precipitation relations across areas with sharp geographic gradients [45] like the near-coastal areas of California. The method was applied to climate simulations spanning the period from 1950–2100, to obtain daily, gridded temperature and precipitation patterns of 21st Century climate over California. Greater detail on the application of the method to California is available in Dettinger [50]. Plots of air temperature and precipitation for the GFDL-A2 and PCM-B1 scenarios are available in Cloern et al. [9].

### Water temperature models

**Fig 2:**
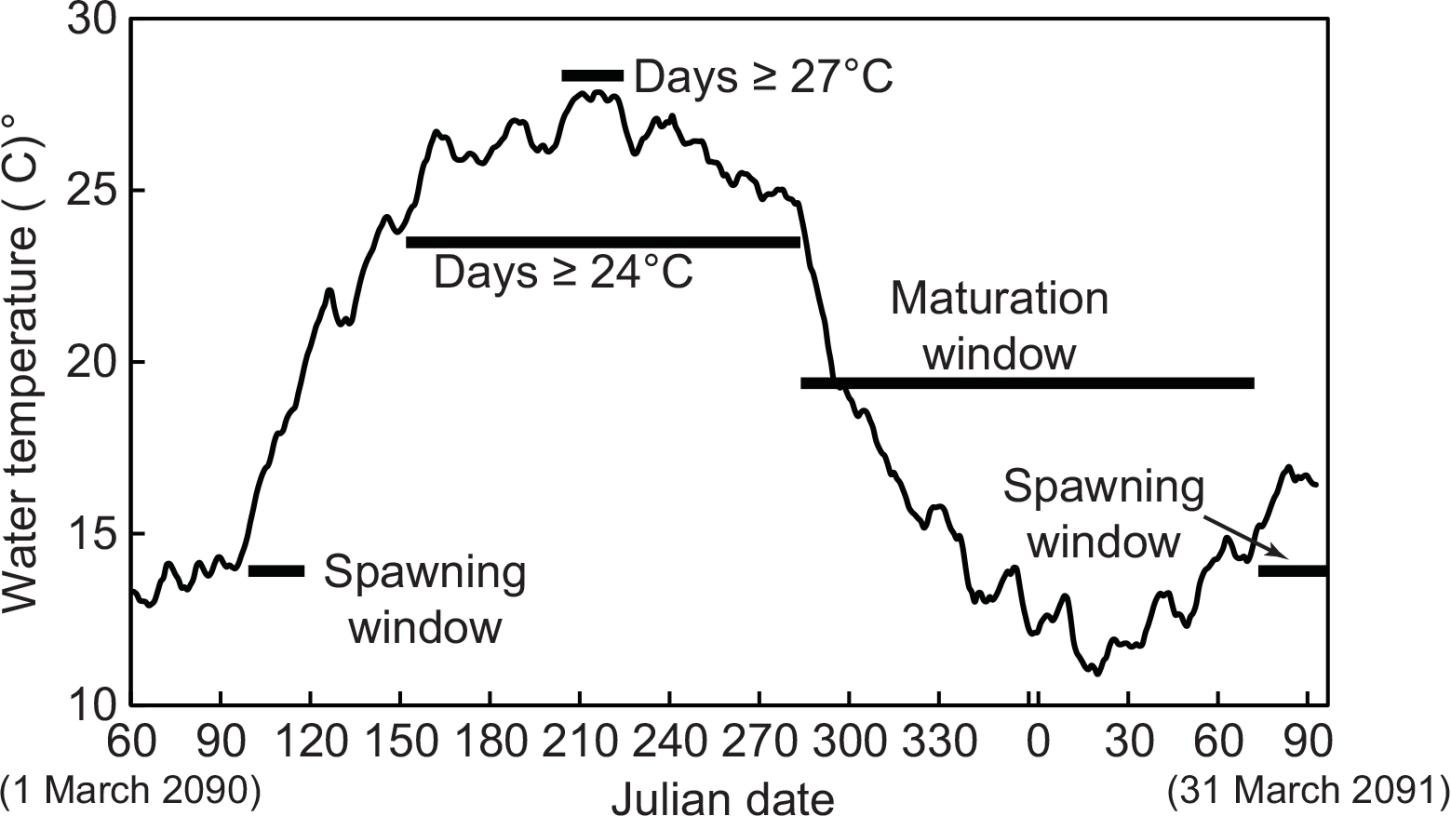
Plot of calculated water temperatures for March 2090 through March 2091. The black bars show the range of days used to calculate the metrics described in the text.

Downscaled average daily air temperatures from the climate change scenarios were sub-sampled for the Delta region, and then averaged to produce Delta daily average air temperature 2000 through 2100 [33]. The climate projections did not provide insolation, so Wagner et al. [33] estimated the average daily insolation from historical data, assuming that insolation will be constant over the century. These data values were then used to generate daily average, minimum, and maximum water temperature for each of the climate change scenarios (Fig 1) as described by Wagner et al. [33]:

$$T(n)=aT_{a}(n)+bT(n-1)+cR(n)+d$$

where *T* represents modeled water temperature, *n* is the day for which the temperature is being calculated, *T*_a_ is the current day’s air temperature, *a* is the coefficient of the current day’s air temperature, *b* is the coefficient of the previous day’s water temperature, *c* is the coefficient on the current day’s insolation, and *d* is a constant offset. Separate models were developed for each site [33]. This modeling approach had high predictive skill at low computational cost. An example plot of a single year of output data from the GFDL-A2 scenario is shown in Fig 2. The models for the locations used in this study performed well with R^2^ >0.93 for calibration and verification data sets. Data were only used from stations with 2 or more years of continuous data [33]. Data were split in half to provide calibration and verification data sets with a minimum of one year of continuous data.

The water temperature data used to calibrate and validate the water temperature models were obtained from the Interagency Ecological Program, which receives the data from the California Department of Water Resources, the United States Geologic Survey, the California Data Exchange Center, and the United States Bureau of Reclamation [33]. Data for the North Delta stations was obtained directly from the United States Geologic Survey. The North Delta stations differed from the other stations in that the temperature sensors varied in depth rather than being 1 m below the surface as for the other stations; however, vertical profiles of water temperature in the North Delta showed no evidence of thermal stratification (S1 Appendix). The largest recorded difference between water temperatures at 1 m and the bottom was 2°C (1 of 116 profiles) with most differences less than 1°C (104 of 116 profiles) (S1 Appendix). Thus, we assume for the purposes of this study that the two sets of stations provide equivalent information and that vertical stratification is likely not an important factor in interpreting our results, though we do not discount them entirely (see Discussion).

Calculated daily mean temperatures used in our analysis are available in S1–S4 Datasets. All temperatures were rounded to the nearest degree. Similarly all metrics described below were rounded to the nearest degree.

### Test organism and metrics

The Delta Smelt is primarily found in the Delta (Fig 1), and is one of the six fish species using the SFE that are currently listed as threatened or endangered under state or federal statutes. Because of its limited geographic range the Delta Smelt may be the most vulnerable to climate change of these fishes. Declining population indices [51,52] have led to concern that the Delta Smelt is in danger of extinction. Like many estuarine species, thresholds of water temperature, salinity, and turbidity are important elements of the physical habitat utilized by Delta Smelt [51,53,54]. The Delta Smelt is primarily an annual species with a small percentage living for 2 years (53). Maturing Delta Smelt move from Suisun, Honker, and Grizzly Bays into the freshwater regions of the Delta during the winter (Fig 1, generally the region including 3–13, except for 6), where they continue to develop. Water temperatures during spawning have not been measured in the wild but larval survival in aquaculture and occurrence of larvae in the estuary suggests that the majority of successful spawning occurs within a spawning window of 15–20°C [53]. After hatching, larval Delta Smelt gradually move seaward toward Suisun Bay [55] as water temperatures in the Delta warm and approach 20°C. Juvenile Delta Smelt rear in the low salinity zone (about 1–6 salinity) or particular freshwater regions. The low salinity zone is generally located from Suisun Bay to the confluence of the Sacramento and San Joaquin Rivers (Fig 1) depending on Delta outflow. Freshwater rearing is concentrated in the northern Delta including the Sacramento River to Rio Vista and then northward to the region around the Sacramento Deepwater Ship Channel [56]. Based on hatchery studies, Delta Smelt growth seems to be optimal at about 20°C with unlimited food [57]. Catches of Delta Smelt in the estuary decrease at locations where temperatures are above 20°C [53,54,58], with Delta Smelt rarely captured at temperatures exceeding 25°C.

We selected metrics for assessing water temperature effects based on the life cycle phenology of the fish and available data on physiological tolerances for the species (see below). Metrics were considered separately for each life stage. Life stages considered included adults (present December-March), post-spawning adults (present March-May), larvae (present March-June), and juveniles (present June-December).

### Chronic lethal thermal maximum (50% mortality)

Komoroske et al. [29] quantified upper thermal acclimation limits using standard chronic thermal tolerance methodology [59]. Chronic Lethal Thermal Maximum (CLT_max_) experiments were conducted for juvenile, adult, and post-spawning adult life stages. Fish were acclimated to laboratory conditions (18.7 ± 0.2°C) for three weeks, and then the temperature was increased by 1°C/d. For each life stage, CLT_max_ was defined as the temperature at which 50% mortality was observed [60,61]. These values of CLT_max_ rounded to the nearest degree were 27°C for both juveniles and adults, and 25°C for post-spawning adults [29]. We chose CLT_max_ as a management relevant indicator of lethal temperatures. It reflects significant but not complete mortality of the population engendered by a relatively gradual increase in temperature compared to other more acute thermal tolerance endpoints, such as critical thermal maxima (CT_max_).

### Onset of physiological thermal stress

Using molecular approaches, Komoroske et al. [62] conducted a series of experiments with hatchery-reared fish to determine temperatures at which physiological thermal stress began for larval and adult Delta Smelt. The sublethal thermal stress thresholds, relative to CT_max_, were found to be consistent across life stages and acclimation temperatures. Thus, for this metric we use the formula *CT*_*max*_ − 4°C to calculate the sublethal physiological stress thresholds for adults and juveniles because these two life stages occur during the summer and fall when warm water temperatures are expected. Since juveniles and adult delta smelt do not differ in their CT_max_ (29), this estimate of the sublethal physiological stress threshold is the same for both stages (24°C for fish acclimated to 16°C). The specific determination of this metric is described below.

Delta Smelt were exposed to varying thermal challenge levels below their critical thermal maximum (*CT*_*max*_ − 6°C, *CT*_*max*_ − 4°C, *CT*_*max*_ − 2°C, control; adult CT_max_ values from Komoroske et al. [29] were 27, 28, and 28°C at acclimation temperatures of 12, 16, and 19°C, respectively) to determine induction thresholds of genes associated with sublethal stress. Sublethal stress can negatively impact organismal performance and fitness below whole organismal tolerance levels [63]. RNA was extracted from gill tissues and analyzed for transcriptomic responses indicative of thermal stress via microarrays and quantitative polymerase chain reaction. Induction of gene transcription indicative of the cellular stress response (CSR; [64]) was most consistently and strongly observed at *CT*_*max*_ − 4°C and *CT*_*max*_ − 2°C across all acclimation temperatures and life stages. However evidence of some level of CSR responses were observed at *CT*_*max*_ − 6°C after 60 minutes of recovery for some groups. These results show that the onset of physiological stress occurs at least 4°C below acute tolerance levels in Delta Smelt (this is a conservative estimate, as stated above, some stress may occur at even lower relative temperatures). For juvenile (CT_max_ of 27, 28, and 29°C at acclimation temperatures of 12, 16, and 19°C, respectively) and adult (CT_max_ presented above) Delta Smelt at medium to high acclimation temperatures, this corresponds with approximately 24°C. Therefore, we used 24°C as a physiological stress onset threshold to quantify the habitat suitability at each downscaled study site.

Note that previous climate change assessments have utilized 25°C thresholds, based on the available data at the time [19]. In this paper, we primarily focus on the juvenile stage in the analysis of the onset of physiological stress because this is the life stage of highest concern with respect to experiencing increasing summer temperatures closest to their thermal tolerance limits [29].

### Spawning window

Increasing water temperatures are likely to affect many aspects of Delta Smelt life history. Both the timing and duration of the spawning window might influence Delta Smelt spawning success. As explained earlier, Delta Smelt spawn in the spring within a temperature window of approximately 15–20°C [53] and we use this temperature range as our definition of the spawning window. We determined two metrics for spawning, the Julian date of the beginning of the spawning window and the duration in days of the spawning window (Fig 2).

### Maturation window

If warm summer temperatures reach stressful levels in the summer and early warming initiates earlier spawning it seems reasonable that there might be less time for juvenile fish to grow before the initiation of maturation and for maturing adults to develop numerous high quality eggs. To evaluate this hypothesis, we define the maturation window as the time period defined by the initiation of daily average water temperatures less than 24°C and the initiation of the spawning window (Fig 2). This represents the period when juvenile fish are able to grow to adulthood and develop mature eggs (or sperm) without interruption by stressful (≥24°C) temperatures. We determined two metrics, the Julian date of the beginning of the maturation window and the duration in days of the maturation window. If the daily average water temperature does not exceed 24°C in a year, then the maturation window is undefined since stressful temperatures never occur. We chose this approach because the length of an uninterrupted maturation window can depend on both the birth date and spawning date of individual fish and both dates can vary from year to year, so assigning a fixed period is difficult to support. We consider our approach conservative because it excludes such pre-defined long windows from analysis.

### Statistical analyses

**Fig 3:**
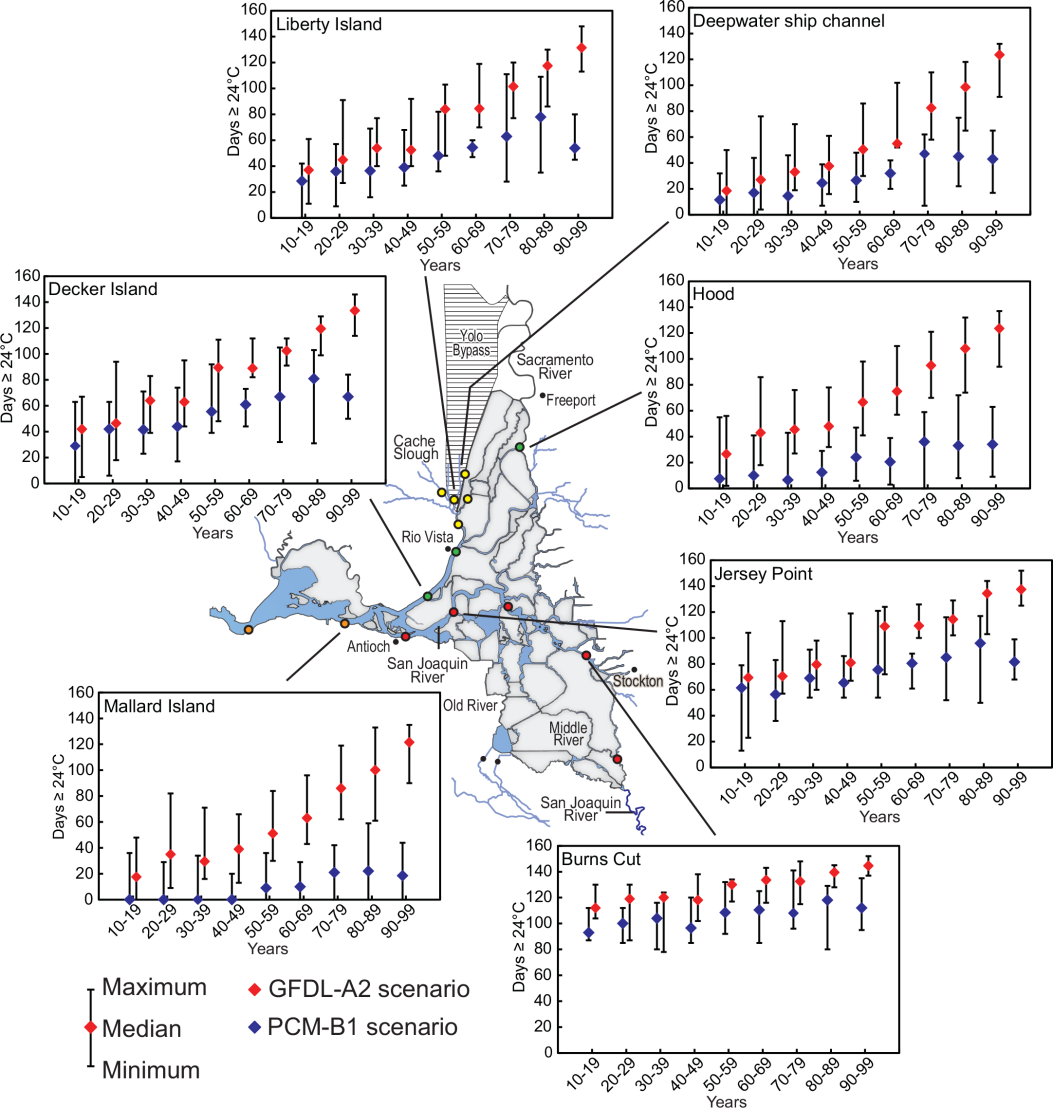
Plots of median, maximum, and minimum for number of days each year with calculated average daily water temperature greater than or equal to 24°C for juvenile Delta Smelt at selected sites during the indicated decades during the 2000s. Results from the warmest scenario (GFDL-A2) and coolest scenario (PCM-B1) considered are shown.

We evaluated trends in the metrics using the Mann-Kendall test [65]. CLT_max_ and onset of physical stress were analyzed as the number of days exceeding the appropriate threshold for each life stage considered and the spawning window and maturation window were analyzed as the number of days within each window. We applied the test to decadal medians of the number of days per year rather than annual values to minimize the effects of large sample size on the determination of significance. Tests were only conducted when 3 or more decadal medians exceeded 0 to ensure we were documenting trends rather than random exceedances of 0. For significant relationships (Mann-Kendall test, P<0.05), we calculated a simple linear regression and used the slope as an approximate indicator of the rate of change. Maximum, minimum and median decadal values for metrics for each life stage and results of the Mann-Kendall test and regression slopes are in supplementary tables (S1–S6 Tables). For graphic presentations, we only show results for the least and most extreme climate change scenarios (PCM-B1 and GFDL-A2, respectively) at selected stations (e.g., Fig 3) for simplicity.

## Results

### Water temperatures

We examined the decadal medians of mean daily water temperatures each year for the juvenile life stage because this life stage includes the summer and fall, which are expected to be the warmest periods. Decadal medians of mean daily water temperatures each year for juveniles increased at all sites in all scenarios; however, the increases in decadal medians were not particularly substantial. For the PCM-B1 scenario, changes were on the order of a single degree (Table 1). Even for the GFDL-A2 scenario, decadal changes in the median of mean daily water temperature each year during the juvenile period were between 2 and 3°C (Table 1). The largest changes for the GFDL-A2 scenario were for the upper two San Joaquin River stations, Mossdale and Burns Cut, with changes of just over 3°C by the end of the century.

**Table 1:** Minimum (Min) and maximum (Max) median decadal value for mean annual daily temperatures during the juvenile life stage of delta smelt (June-December) for the least-warming (PCM-B1) and most-warming (GFDL-A2) climate change scenarios. The difference between the minimum and maximum is also shown.

	PCM-B1			GFDL-A2		
	Min	Max	Difference	Min	Max	Difference
**San Joaquin River**						
Mossdale	20.2	21.4	1.2	20.3	23.4	3.1
Burns Cut	21.1	22.3	1.2	21.2	24.4	3.2
Prisoners Point	19.7	20.7	1.0	19.8	22.4	2.6
Jersey Point	20.1	21.3	1.1	20.2	23.2	2.9
Antioch	19.7	20.8	1.1	19.8	22.6	2.8
**Sacramento River**						
Hood	18.7	19.8	1.1	18.9	21.7	2.8
Rio Vista	19.1	20.2	1.1	19.2	22.1	2.9
Decker Island	19.7	20.8	1.1	19.8	22.7	2.9
**North Delta**						
Upper Cache Slough	18.5	19.5	1.0	18.6	21.2	2.6
Miners Slough	18.6	19.6	1.0	18.7	21.3	2.5
Liberty Island	19.3	20.4	1.1	19.4	22.3	2.9
Deepwater Ship Channel	18.9	19.9	1.0	19.0	21.6	2.6
Lower Cache Slough	18.8	19.8	1.0	18.9	21.6	2.6
**Confluence**						
Mallard Island	18.9	19.9	1.1	19.0	21.7	2.7
**Suisun Bay**						
Martinez	18.4	19.4	1.0	18.5	21.0	2.5

### Chronic lethal thermal maximum (50% mortality)

Decadal median number of days each year ≥ CLT_max_ never exceeded zero for adults for any scenario. Medians for post-spawning adults exceeded zero but these temperatures occurred after the spawning window had ended.

Juveniles were the life stage that experienced exceedances of CLT_max_ most often. For the PCM-B1 scenario, decadal median number of days each year ≥CLT_max_ rarely exceeded zero, except at the two most upstream sites on the San Joaquin (S1 Table). At Burns Cut values ranged from 41.5 days in the 2010s to 90.5 days in the 2080s and at Mossdale values ranged from 21.5 in the 2010s to 63 days in the 2080s; however, these locations are already rarely inhabited by Delta Smelt under current conditions. Results were similar for the intermediate scenarios with the decadal median number of days each year ≥CLT_max_ increasing to greater than zero at stations other than Mossdale and Burns Cut but never exceeding 20 days at any station by the end of the century, except for Antioch. Under the GFDL-A2 scenario, decadal median number of days each year ≥CLT_max_ exceeded zero by midcentury and exceeded 50 days by the end of the century at most stations in the San Joaquin and Sacramento Rivers and the North Delta (S1 Table).

### Onset of physiological thermal stress

Stressful temperatures occurred most often during the juvenile life stage. There were substantial differences among the scenarios in the number of days juvenile Delta Smelt experienced stressful temperatures. By the end of the century the decadal median number of days each year ≥24°C exceeded 60 at most stations under the intermediate scenarios PCM-A2 and GFDL-B1 and exceeded 100 days at most stations under the GFDL-A2 scenario (S2 Table). All trends were statistically significant and slopes varied from about 2 to 6 days per decade for the PCM-B1 scenario and from about 4 to 12 days per decade for the GFDL-A2 scenario (S2 Table). When the data are viewed geographically, it is clear that a large part of the Delta will become physiologically stressful for Delta Smelt for extended periods of time as climate change proceeds (Fig 3). It is noteworthy that the wide range in metric values during each decade (Fig 3) suggests that stressful temperatures are possible for extended periods well before the decadal median value becomes large. Conversely, low values are also possible later in the century (Fig 3).

### Spawning window

Significant trends in duration of the spawning window were only observed for the GFDL-A2 scenario (S3 Table). The significant trends indicate that the duration of the spawning window may be longer at some sites by the end of the century, but the rates of change are modest, from 1 to 3 days per decade. There were significant trends in the beginning date of the spawning window for some sites for the PCM-A2 and GFDL-B1 scenarios and at all sites for the PCM-B1 and GFDL-A2 scenarios (Table 2; S4 Table). Trends were all negative indicating earlier initiation of spawning. For scenarios other than GFDL-A2, rates of change varied between about 1 and 2 days per decade. For GFDL-A2, rates were higher, ranging from 3 to 5 days per decade (Table 2). The maximum difference in beginning date (latest date—earliest date) ranged from 20–41 days for the four scenarios. The highest rates of change occurred in the North Delta for the GFDL-A2 scenario, with results for the other scenarios less clear (Table 2).

**Table 2:** Minimum (Min) and maximum (Max) of median decadal value for julian date of the beginning of the spawning window (15–20°C) each year, during the century (2010–2099) for the least-warming (PCM-B1), most-warming (GFDL-A2) and two intermediate (PCM-A2 and GFDL-B1) climate change scenarios. The significance value for Trend is from the Mann-Kendal test (NS, P>0.05; *, P<0.05; **, P<0.01; ***, P<0.001; NA, no non-zero values) and the number is the slope of a regression of decadal medians.

	PCM-B1			PCM-A2			GFDL-B1			GFDL-A2		
	Min	Max	Trend	Min	Max	Trend	Min	Max	Trend	Min	Max	Trend
**San Joaquin River**												
Mossdale	74.5	88.0	-1.28*	68.0	86.5	-2.22*	69.5	91.5	-2.09*	49.0	81.0	-3.56**
Burns Cut	79.5	90.0	-1.29**	73.0	92.0	-1.96**	76.5	93.0	NS	65.0	88.0	-2.57**
Prisoners Point	78.5	90.0	-1.27**	73.0	88.5	-1.95**	76.0	93.5	NS	64.0	86.5	-2.82**
Jersey Point	86.5	100.0	-1.44***	82.5	102.5	NS	82.5	103.0	-1.87*	70.5	98.5	-3.58**
Antioch	82.5	94.5	-1.63**	76.5	96.5	-2.17**	77.0	96.0	NS	64.0	91.0	-3.56**
**Sacramento River**												
Hood	89.5	101.5	-1.19**	84.0	110.0	-1.65*	93.0	103.5	-1.19**	79.5	103.0	-2.98**
Rio Vista	89.0	101.5	-1.23**	83.5	110.0	NS	92.5	103.5	-1.23*	78.5	103.0	-3.06**
Decker Island	91.5	105.0	-1.23**	85.0	111.5	-1.88*	94.0	105.5	-1.18*	79.0	104.5	-3.44**
**North Delta**												
Upper Cache Slough	84.5	100.5	-1.38**	48.0	101.0	NS	77.0	101.5	-2.23**	56.5	96.5	-4.78**
Miners Slough	85.5	100.5	-1.38***	82.0	102.0	NS	78.5	102.5	-2.01*	69.0	98.0	-4.09***
Liberty Island	85.5	100.5	-1.43***	82.0	102.0	NS	83.0	103.0	-1.84*	59.5	100.5	-4.92**
Deepwater Ship Channel	83.5	97.0	-1.68***	80.0	101.0	NS	76.5	98.5	-1.93*	54.5	95.5	-4.73**
Lower Cache Slough	84.0	100.0	-1.83***	80.0	101.0	NS	77.0	100.5	-2.31*	56.0	96.5	-4.74**
**Confluence**												
Mallard Island	87.5	101.0	-1.42***	83.0	110.0	NS	86.0	103.0	NS	72.5	99.5	-3.53**
**Suisun Bay**												
Martinez	88.5	101.5	-1.28**	83.5	110.5	NS	90.0	104.5	-1.41*	75.5	103.5	-3.6**

### Maturation window

Because the maturation window metric is defined as beginning in the summer or fall when modeled water temperatures decline below 24°C, the maturation window is undefined if water temperatures did not reach 24°C that year. This situation occurred most often at Martinez, for the PCM-B1 and PCM-A2 scenarios (S5 Table). Except for Martinez, all sites had a maturation window defined in at least 8 of 10 years by mid-century. For all scenarios except PCM-B1, there were maturation windows defined in at least 7 years in every decade of the study period and most sites had a maturation window every year (S5 Table).

There were significant trends in the beginning of the maturation window for the majority of the stations (S5 Table). Exceptions were Martinez, Burns Cut and Mossdale for PCM-B1, Martinez for PCM-A2, and Martinez for GFDL-A2. Minimum decadal median beginning dates ranged from mid-July to mid-September across all sites and scenarios, and maximum decadal median beginning dates ranged from September to mid-October across all sites and scenarios. Rates of change for significant trends were all positive indicating later initiation of the maturation window and ranged from about 1–4 days later per decade for PCM-B1, 2–6 days later for PCM-A2, 1–5 days later for GFDL-B1, and 6–12 days later per decade for GFDL-A2.

**Fig 4:**
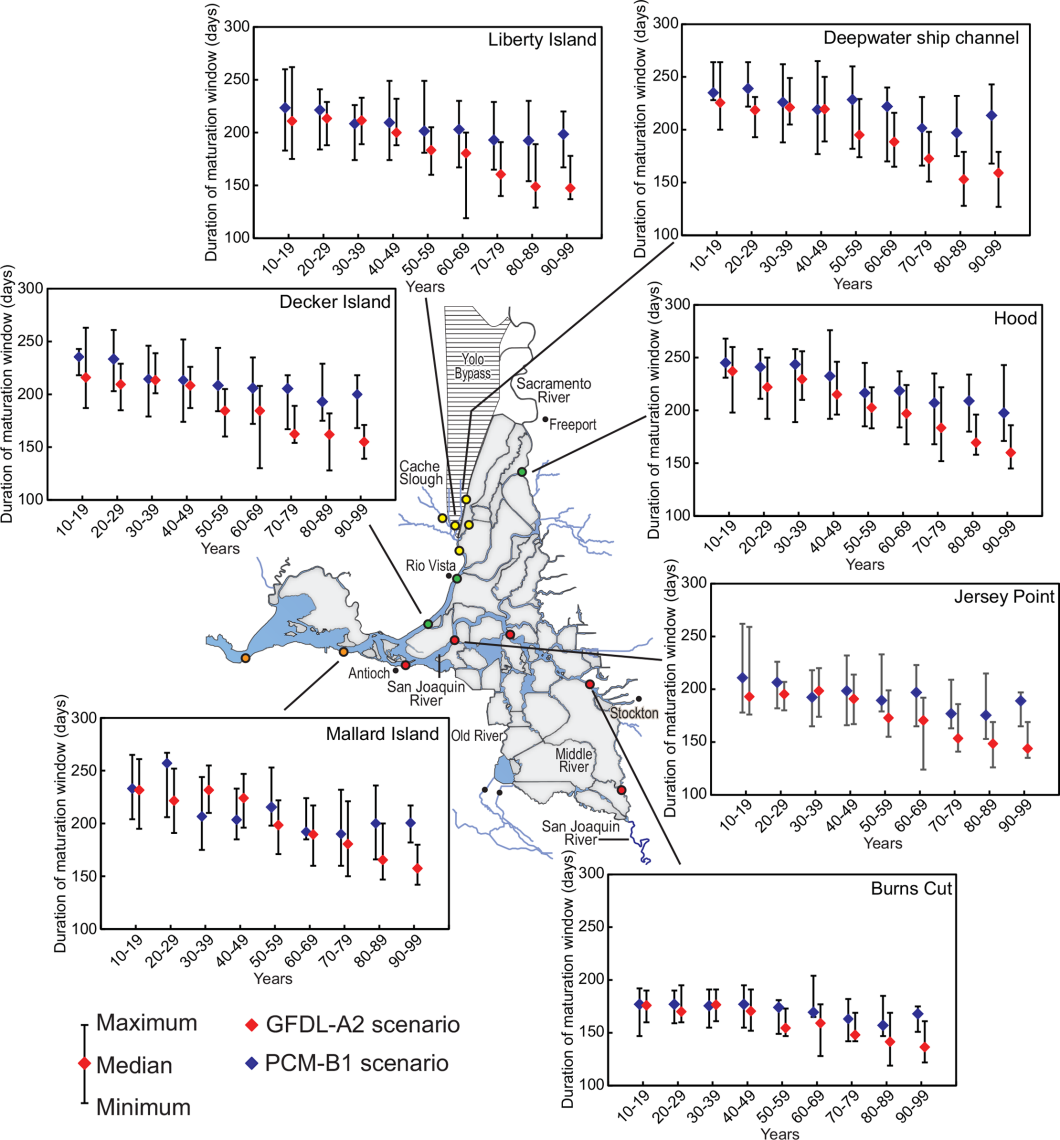
Plots of median, maximum, and minimum for number of days each year with calculated average daily water temperature within the maturation window for juvenile Delta Smelt at selected sites during the indicated decades during the 2000s. Results from the warmest scenario (GFDL-A2) and coolest scenario (PCM-B1) considered are shown.

There were substantial changes in the duration of the spawning window (S6 Table). There was not a clear declining trend for the all the scenarios until mid-century (Fig 4). PCM-B1 exhibited the smallest changes with differences between the longest decadal median duration and the shortest subsequent duration ranging from 18 to 48 days, depending on station (S6 Table). The San Joaquin River stations appeared to show the smallest changes for all scenarios (Fig 4, S6 Table). GFDL-A2 exhibited the largest changes with differences between the longest decadal median duration and the shortest subsequent duration ranging from 40 to 85 days (Fig 4, S6 Table). Rates of change for significant trends were all negative indicating shorter duration of the maturation window and ranged from about 2–6 days shorter per decade for PCM-B1, 4–7 days shorter for PCM-A2, 3–6 days shorter for GFDL-B1, and 5–12 days shorter per decade for GFDL-A2 (S6 Table).

## Discussion

Our strategy of coupling spatially explicit climate change models with knowledge of life history and physiology offers critical insight into understanding habitat suitability for Delta Smelt under multiple climate change scenarios. Our results suggest that even the intermediate-high degree of climate change represented by our GFDL-A2 scenario will likely have important effects on the Delta Smelt population. Although lethal temperatures do occur under the scenarios of climate change examined (S1 Table), the occurrence of sublethal stressful temperatures (S2 Table) is likely more important ecologically. Freely-moving organisms like pelagic fishes will frequently respond to stressful environmental cues behaviorally, and leave before conditions become lethal [30]. Although Delta Smelt are not very strong swimmers [66], juvenile Delta Smelt are capable of rapid movement in the estuary by controlling their position in the water column to take advantage of tidal water movements [67,68]. Presumably, juveniles will move out of regions that become stressful. Movements out of the southern Delta (south of the San Joaquin River) in the spring have already been noted and associated with water temperature and increasing water clarity [58]. Much of the habitat in the Delta becomes progressively less hospitable as climate change proceeds and presumably Delta Smelt will spend more time in Suisun Bay, which will remain relatively cooler (S2 Table). Since Delta Smelt are unlikely to undergo a range shift (i.e., migrate into open coastal Pacific waters to seek estuarine habitat at northern latitudes), the loss of suitable thermal habitat in the Delta represents significant habitat compression for this species. Our results suggest that there will likely be significant thermal habitat constriction for Delta Smelt over the next 50 years.

The shortening of the maturation window is of particular concern because of its possible effects on reproductive potential of the Delta Smelt population. Fecundity of Delta Smelt is a function of fish length [69], similar to many other fishes, so the growth and nutrition, which affects egg quality, of juvenile fish is important. If we assume the juvenile life stage lasts from June through December (113 days), our results suggest loss of 16 to 43% of the maturation window over the century for the PCM-B1 scenario and of 35 to 75% over the century for the GFDL-A2 scenario, depending on station. Overall, the Delta will become less and less hospitable to Delta Smelt for increasing lengths of time as climate change proceeds. This is particularly sobering because the lower Sacramento River and North Delta represent a large portion of the Delta Smelt’s range, and substantial habitat restoration is planned in the North Delta intended, in part, to benefit Delta Smelt.

Occurrence of stressful days during the juvenile life stage combined with earlier initiation of the spawning window decreased the number of days available for maturation under favorable physiological conditions. Although we do not have direct evidence, it is likely that these conditions could induce negative maternal effects [70], such as reduction in total egg production or quality in the population, or other impacts on performance, and consequently, fitness. Individual fecundity of Delta Smelt and many other fishes is directly related to length [53,71], which may be reduced either due to lower growth rates related to physiological stress costs or via truncated maturation windows. Both the number and quality of eggs can be influenced by interactions of temperature and feeding rates and the costs of stress can reduce the quality of gametes produced [72]. Additionally, while the majority of Delta Smelt die after their first spawning season, the small percentage that are able to survive to spawn in a second year have substantially higher reproductive output [53]. However, the decreased thermal tolerance of post-spawning adults [29] coupled with increasing summer temperatures may effectively eliminate these individuals, further decreasing overall fecundity in the population.

It is also important to note that phenology of life stages plays a large role in the estimated exposure of specific Delta Smelt life stages to stressful thermal habitat conditions. The current relationships may change with the shifting of maturation windows, earlier spawning windows, or other impacts. We did not explicitly assess the occurrence of stressful temperatures for adult Delta Smelt because daily mean water temperatures were not predicted to exceed the 24°C threshold during the months coinciding with adult presence according to the current Delta Smelt life cycle, but this may change in the future. Additionally, our spawning window calculations were based solely on temperature projections. However, it is widely thought that Delta Smelt spawning cues include migration in response to ‘first flush’ rain storm events in the late fall or early winter months, and timing and severity of rainstorms are also likely to be affected by climate change. Therefore, timing shifts could result in the mismatching of cues, a phenomenon that has been documented in many other species [73], and could further negatively affect reproductive success. In addition to phenological changes, genetic adaptation may temper negative impacts of warming via the evolution of increased thermal tolerance in some species [74]. Since Delta Smelt have a relatively short lifespan, such rapid evolution may be possible, potentially lessening the thermal habitat reduction predicted by our study. However, Delta Smelt exhibit limited acclimation capacity [62] and little variation among individuals in the population in thermal phenotypes (i.e., critical thermal limits) [29]. Such low thermal plasticity and functional genetic variation may restrict species’ ability to rapidly respond to changes in environmental selection pressures [74,75], suggesting that Delta Smelt may have little potential for rapid thermal adaptation. However, this has yet to be investigated and currently remains unresolved for this species. Both phenological and rapid adaptation issues underscore the importance of considering both direct and indirect effects of climate change on species of interest, and incorporating new information into evaluations if either environmental conditions or relevant species biology changes.

Habitat that remains thermally adequate may be sub-optimal for other important ecological reasons and the interactions of multiple environmental stressors may affect population distribution and abundance. For example, it is plausible that Delta Smelt would seek refuge in typically cooler, lower SFE habitats. However, as water temperatures increase with climate change, increased salinity intrusion is also expected as a result of sea-level rise [9]. Therefore, although Suisun Bay may have the most favorable water temperatures, salinity is likely to increase. Delta Smelt are capable of acclimating to salinities as high as seawater for at least a month [29] but there is some evidence that this imposes physiological costs that currently make high salinities sub-optimal [76]. The ability to adjust physiological strategies to deal with large changes in salinity resulting from seasonal cycles, life history related movements or direct environmental changes [77], is reasonably common among osmerids [78,79]. If future suitable thermal habitat coincided with higher salinities, it is possible that selection pressures could result in Delta Smelt acclimatizing or adapting to meet the challenges of these conditions. However, covariation of salinity and other factors in the SFE (e.g., turbidity and possibly food resources; [67,69]) constrain a comprehensive understanding of Delta Smelt’s current ecological association with low salinity waters. Interactions of multiple stressors may play important roles in Delta Smelt’s ability to exploit cooler, saltier habitat in the future [24,25]. Clearly, the complexity of estuarine systems makes it difficult to assess the future effects of climate change based on a single factor; however, water temperature is a key habitat characteristic for fishes [35,36]. Given that other environmental conditions affecting Delta Smelt abundance and distribution, such as turbidity and food availability, are also becoming less favorable [67,69], our assessment is likely conservative. We also note that the temperature models developed by Wagner et al. [33] are statistical models and large changes in Delta configuration, such as flooding of large areas of current agricultural land or re-routing of flows through canals or pipes, could affect the accuracy of calculated future temperatures. A process-based temperature model linked to a hydrodynamic model is needed to avoid such problems.

Our modeling results have important management implications because water management operations in California are often closely tied to populations of organisms of conservation interest. In the Delta, management of Delta Smelt is a major driver in the operations of the Central Valley Project and California State Water Project. Habitat restoration to enhance Delta Smelt habitat directly or indirectly through food web augmentation has been proposed (e.g., Bay Delta Conservation Plan; [31]). One area targeted for extensive restoration efforts is the North Delta, with both Liberty Island and the Deepwater Ship Channel identified because Delta Smelt can currently inhabit this area over the entire year. Our results suggest that such restoration may not be fully sustainable because restored areas are unlikely to be accessible to Delta Smelt over the entire year as climate change proceeds. The possible indirect benefits of food web augmentation for downstream populations is unclear given unknown consumption rates by other organisms between the North Delta and the confluence. We do not fully discount the possibility of temperature refugia because our models do not incorporate vertical or lateral variability in temperature; however, the data we have available suggest that such refugia would be limited (S1 Appendix). We also acknowledge that our statistical models need to be recalibrated if major changes in infrastructure or water operations take place. Because Delta Smelt move within the SFE, their presence within the estuary differs spatially through their life cycle. The distribution and timing of life stages has already changed, as juvenile habitat in the southern Delta has contracted from historical conditions [58,80]. Providing managers with the locally projected thermal habitat suitability for each location, offers insight into the likelihood (or lack thereof) of future habitat shifts as Delta Smelt respond to climate change in coming decades.

Balancing the protection of species with human water needs is difficult at the best of times. As climate change alters precipitation, temperature, and sea-level, society will be forced to prioritize which species or ecosystems should be conserved. Delta Smelt and Winter-run Chinook Salmon are endemic organisms only found in the SFE and its watershed. These species have been given protection under the federal and state endangered species act, even though the societal and economic issues are difficult to address [81,82]. New approaches to understanding species responses to climate change and linking those responses to management at relevant scales will continue to improve our ability to bring science to these issues. Conducting climate change assessments over smaller spatial scales not only addresses knowledge gaps of forecasted climate change impacts, but also provides managers and scientists with critical information to incorporate into conservation plans. As downscaled GCMs continue to improve, this approach can serve as a useful tool to identify and mitigate climate change effects in ecosystems with baseline data on abiotic conditions and species’ biology.

## Supporting Information

S1 Appendix Methods and results for measurements of north Delta water temperature. (PDF)

S1 Dataset Calculated water temperatures for the PCM-B1 climate change scenario. (XLSX)

S2 Dataset Calculated water temperatures for the PCM-A2 climate change scenario. (XLSX)

S3 Dataset Calculated water temperatures for the GFDL-B1 climate change scenario. (XLSX)

S4 Dataset Calculated water temperatures for the GFDL-A2 climate change scenario. (XLSX)

S1 Table Median, minimum, and maximum values for the number of days each year when mean daily water temperature is ≥CLT_max_, during each decade from 2010–2099, for the juvenile life stage of Delta Smelt (June-December) for the least-warming (PCM-B1), most-warming (GFDL-A2) and two intermediate (PCM-A2 and GFDL-B1) climate change scenarios. (PDF)

S2 Table Median, minimum, and maximum values for the number of days per year when mean daily water temperature is ≥24°C, during each decade from 2010–2099, for the juvenile life stage of Delta Smelt (June-December) for the least-warming (PCM-B1), most-warming (GFDL-A2) and two intermediate (PCM-A2 and GFDL-B1) climate change scenarios. (PDF)

S3 Table Median, minimum, and maximum values for the number of days per year for the duration of the spawning window (15–20°C), during each decade from 2010–2099, for the adult life stage of Delta Smelt for the least-warming (PCM-B1), most-warming (GFDL-A2) and two intermediate (PCM-A2 and GFDL-B1) climate change scenarios. (PDF)

S4 Table Median, minimum, and maximum values for the julian date of the beginning of the spawning window (15–20°C) each year, during each decade from 2010–2099, for the adult life stage of Delta Smelt for the least-warming (PCM-B1), most-warming (GFDL-A2) and two intermediate (PCM-A2 and GFDL-B1) climate change scenarios. (PDF)

S5 Table Median, minimum, and maximum values for the julian date of the beginning of the maturation window (last day of 24°C to beginning of the spawning window) each year during each decade from 2010–2099, for the adult life stage of Delta Smelt for the least-warming (PCM-B1), most-warming (GFDL-A2) and two intermediate (PCM-A2 and GFDL-B1) climate change scenarios. (PDF)

S6 Table Median, minimum, and maximum values for the number of days per year for the duration of the maturation window (last day of 24°C to beginning of the spawning window) during each decade from 2010–2099, for the adult life stage of Delta Smelt for the least-warming (PCM-B1), most-warming (GFDL-A2) and two intermediate (PCM-A2 and GFDL-B1) climate change scenarios. (PDF)
