# Supplementary material for: Coupled Downscaled Climate Models and Ecophysiological Metrics Forecast Habitat Compression for an Endangered Estuarine Fish
Source: PLoS One. 2016 Jan 21;11(1):e0146724. doi: 10.1371/journal.pone.0146724 (PMC4721863; doi:10.1371/journal.pone.0146724)
Supplement: S1 Appendix — (PDF) [file pone.0146724.s001.pdf]

S1 Appendix. Methods and results for measurements of north Delta water temperature

Because of differences in sensor depth between the North Delta water temperature stations (Table 1) and the other water temperature stations (depth of 1 m) used in this study, we measured vertical profiles of water temperature at a number of locations in the North Delta (Fig 1). Our purpose was to determine if the different sensor depths would likely produce data that would introduce large biases into our water temperature models. We also wanted to determine if persistent thermal stratification occurred in the North Delta or the Sacramento River, which might provide a possible temperature refuge for our test organism, Delta Smelt. Temperatures were measured primarily with a YSI EXO2 multiparameter sonde with a temperature response of less than 1 second. An RBR concerto Conductivity-Temperature-Depth profiling unit with temperature response of 0.1 second was used in a few cases. Limited side-by-side comparisons indicated no differences in measured water temperatures between the two instruments.

Table 1. Site names from main article, average depth of sensors for North Delta locations where water temperature models were developed, and nearest location where water temperature profiles were collected (see Fig 1).

Site name	Average depth of sensor (m)	Nearest vertical profile location
Miners Slough	3.7	11
Sacramento River deepwater ship channel	1.6	56, 62
Lower Cache Slough	2.7	44
Upper Cache Slough	4.5	5
Liberty Island	1.8	1

Examination of data for water temperature at 1m below the surface and at the bottom of the water column indicated little difference in temperature (Table 2). At most locations, differences were less than 1°C. The largest differences (>1°C) occurred in the Deepwater ship channel and Shag Slough in the early afternoon during August and September. The Deepwater ship channel is a deep navigation channel that dead ends at the Port of Sacramento and Shag Slough is near the upper end of tidal influence in the North Delta. The absence of large differences between surface and bottom temperatures, even in areas with muted tidal mixing is expected, suggests that thermal stratification is not a regular feature in the North Delta and that our models are likely adequate for estimating future water temperatures. In addition, it seems unlikely that Delta Smelt would be able to find substantial relief from warm surface temperatures by moving deeper in the water column.

Table 2. Site number (Fig 1), date, start time, depth, and water body for vertical profiles of water temperature. Site numbers 16-84 refer to navigational channel markers along the Sacramento River, lower Cache Slough, and the Deepwater ship channel (see S1_Fig 1). The values are for water temperature at 1m depth, water temperature at the bottom, and the difference between them.

Site number	Date	Start time	Water body name	Depth (m)	Water temper-ature at 1 m (°C)	Water temper-ature at bottom (°C)	Differ-ence (°C)
1	5/14/2014	9:38 AM	Liberty Island	13.0	20.3	20.3	0.0
1	5/28/2014	12:32 PM	Liberty Island	1.6	20.5	20.2	0.3
1	5/28/2014	12:35 PM	Liberty Island	11.4	20.4	20.1	0.3
1	6/18/2014	13:30 PM	Liberty Island	13.7	21.6	21.6	0.0
1	7/8/2014	13:45 PM	Liberty Island	13.0	24.0	24.1	0.0
2	5/14/2014	8:52 AM	Lindsey Slough	3.8	20.1	20.0	0.1
2	5/28/2014	12:09 PM	Lindsey Slough	3.8	21.0	20.8	0.2
3	7/8/2014	13:19 PM	Upper Cache Slough/Shag Slough	10.2	23.6	23.6	0.0
4	7/8/2014	12:53 PM	Shag Slough	8.4	23.5	23.5	0.0
5	4/8/2014	13:10 PM	Upper Cache Slough	5.4	18.7	18.1	0.6
5	4/24/2014	10:05 AM	Upper Cache Slough	5.4	17.8	17.6	0.2
5	5/14/2014	9:15 AM	Upper Cache Slough	4.7	21.2	19.4	1.8
5	6/18/2014	13:10 PM	Upper Cache Slough	5.3	21.9	20.6	1.3
5	7/8/2014	12:20 PM	Upper Cache Slough	5.6	23.4	22.4	1.0
5	8/26/2014	11:53 AM	Upper Cache Slough	3.7	20.7	20.2	0.5
5	5/28/20104	11:45 AM	Upper Cache Slough	4.9	20.5	20.4	0.1
6	8/21/2014	1:47 PM	Shag Slough	5.5	22.3	21.2	1.1
6	8/26/2014	12:14 PM	Shag Slough	6.0	21.5	20.8	0.7

6	8/26/2014	12:36 PM	Shag Slough	6.9	21.5	20.8	0.7
6	8/26/2014	12:55 PM	Shag Slough	6.8	21.3	20.8	0.5
6	8/26/2014	14:33 PM	Shag Slough	7.4	22.8	20.8	2.0
6	9/18/2014	10:02 AM	Shag Slough	7.4	21.8	21.5	0.4
6	9/18/2014	11:23 AM	Shag Slough	7.8	21.9	21.6	0.3
6	9/18/2014	13:28 PM	Shag Slough	6.9	22.3	22.1	0.3
6	9/18/2014	13:39 PM	Shag Slough	7.0	22.5	22.1	0.4
6	9/18/2014	13:55 PM	Shag Slough	7.1	22.4	22.0	0.4
6	9/18/2014	14:11 PM	Shag Slough	7.3	22.3	21.9	0.4
6	9/18/2014	14:27 PM	Shag Slough	7.2	22.4	21.8	0.6
6	9/18/2014	14:41 PM	Shag Slough	7.2	22.3	21.8	0.5
6	9/18/2014	14:57 PM	Shag Slough	7.1	22.3	21.8	0.5
7	7/8/2014	11:35 AM	Shag Slough	4.1	23.2	22.9	0.3
7	8/26/2014	13:15 PM	Shag Slough	6.1	22.4	20.9	1.5
7	8/26/2014	13:35 PM	Shag Slough	6.7	22.5	20.9	1.6
7	8/26/2014	14:18 PM	Shag Slough	6.6	22.6	20.9	1.7
8	8/26/2014	14:00 PM	Stairstep	6.2	21.4	21.3	0.1
9	6/18/2014	12:16 PM	Prospect Slough/Liberty Cut	4.6	21.1	20.7	0.3
9	7/8/2014	9:24 AM	Prospect Slough/Liberty Cut	4.6	22.9	22.8	0.1
9	7/8/2014	9:31 AM	Prospect Slough/Liberty Cut	4.8	23.2	22.8	0.3
9	7/8/2014	9:50 AM	Prospect Slough/Liberty Cut	6.9	23.1	22.9	0.3
9	7/8/2014	10:15 AM	Prospect Slough/Liberty Cut	6.5	23.0	23.0	0.0
9	7/8/2014	10:57 AM	Liberty Cut	6.6	23.1	23.0	0.1
10	5/28/2014	13:01 PM	Toe Drain	4.6	20.8	20.6	0.1
10	6/18/2014	12:40 PM	Toe Drain	4.8	21.2	21.1	0.1
11	5/28/2014	9:55 AM	Miners Slough	6.6	23.0	23.0	0.1
12	4/9/2014	14:08 PM	Miners Slough	6.3	17.7	17.2	0.5
12	4/24/2014	11:33 AM	Miners Slough	6.7	19.8	19.7	0.1
12	5/14/2014	12:44 PM	Miners Slough	5.7	21.6	21.6	0.1

12	6/18/2014	9:39 AM	Miners Slough	7.3	22.3	22.3	0.0
16	8/26/2014	8:34 AM	Sacramento River, Decker Island	9.1	21.3	21.3	0.0
16	10/21/2014	8:06 AM	Sacramento River, Decker Island	8.4	19.6	19.6	0.0
16	10/23/2014	7:51 AM	Sacramento River, Decker Island	7.8	19.3	19.3	0.0
34	8/26/2014	9:08 AM	Sacramento River, Rio Vista	9.4	22.1	22.1	0.0
34	10/21/2014	8:51 AM	Sacramento River, Rio Vista	9.2	19.7	19.7	0.0
34	10/23/2014	8:14 AM	Sacramento River, Rio Vista	6.4	19.3	19.3	0.0
44	4/9/2014	14:46 PM	Lower Cache Slough	15.1	16.7	16.7	0.0
44	4/24/2014	13:48 PM	Lower Cache Slough	15.6	19.5	19.3	0.2
44	5/14/2014	8:24 AM	Lower Cache Slough	15.8	20.2	20.2	0.0
44	5/28/2014	13:25 PM	Lower Cache Slough	14.7	21.2	20.8	0.4
44	6/18/2014	13:52 PM	Lower Cache Slough	15.1	21.9	21.8	0.1
44	7/8/2014	14:06 PM	Lower Cache Slough	16.0	24.3	24.2	0.1
44	8/26/2014	9:42 AM	Lower Cache Slough	14.3	21.6	21.4	0.2
44	10/21/2014	9:19 AM	Lower Cache Slough	11.7	19.2	19.2	0.0
44	10/23/2014	8:42 AM	Lower Cache Slough	11.6	19.0	19.0	0.0
54	4/9/2014	13:36 PM	Sacramento River deepwater ship channel	10.6	17.7	17.0	0.7
54	4/24/2014	12:15 PM	Sacramento River deepwater ship channel	11.3	19.3	19.1	0.2
54	5/14/2014	12:02 PM	Sacramento River deepwater ship channel	10.1	20.7	20.1	0.6
54	5/28/2014	10:13 AM	Sacramento River deepwater ship channel	10.6	21.4	21.3	0.1
54	6/18/2014	11:21 AM	Sacramento River deepwater ship channel	10.6	21.5	21.4	0.1
54	7/7/2014	15:11 PM	Sacramento River deepwater ship channel	10.6	23.9	23.3	0.6
54	8/26/2014	10:17 AM	Sacramento River deepwater ship channel	10.4	21.8	21.6	0.2
56	8/26/2014	10:39 AM	Sacramento River deepwater ship channel	10.7	21.9	21.7	0.2
56	10/21/2014	9:52 AM	Sacramento River deepwater ship channel	10.7	19.6	19.5	0.1
56	10/23/2014	9:56 AM	Sacramento River deepwater ship channel	10.5	19.4	19.4	0.0
62	8/26/2014	11:06 AM	Sacramento River deepwater ship channel	9.4	21.8	21.3	0.5
62	10/21/2014	10:34 AM	Sacramento River deepwater ship channel	7.3	19.6	19.5	0.1
62	10/23/2014	10:18 AM	Sacramento River deepwater ship channel	8.1	19.4	19.3	0.1

66	5/14/2014	11:22 AM	Sacramento River deepwater ship channel	9.2	20.2	19.7	0.5
66	5/28/2014	10:51 AM	Sacramento River deepwater ship channel	9.8	21.3	21.2	0.1
66	6/18/2014	10:41 AM	Sacramento River deepwater ship channel	10.5	21.3	21.1	0.3
66	6/26/2014	11:03 AM	Sacramento River deepwater ship channel	7.8	21.9	21.7	0.2
66	6/26/2014	11:15 AM	Sacramento River deepwater ship channel	9.0	21.9	21.7	0.2
66	6/26/2014	11:30 AM	Sacramento River deepwater ship channel	9.6	22.0	21.8	0.3
66	6/26/2014	11:46 AM	Sacramento River deepwater ship channel	7.0	22.0	21.8	0.2
66	6/26/2014	11:49 AM	Sacramento River deepwater ship channel	9.4	22.1	21.8	0.3
66	6/26/2014	12:01 PM	Sacramento River deepwater ship channel	9.0	22.1	21.9	0.3
66	6/26/2014	12:17 PM	Sacramento River deepwater ship channel	9.1	22.2	21.8	0.4
66	6/26/2014	12:22 PM	Sacramento River deepwater ship channel	8.8	22.1	21.8	0.3
66	6/26/2014	12:34 PM	Sacramento River deepwater ship channel	8.8	22.2	21.8	0.4
66	6/26/2014	12:45 PM	Sacramento River deepwater ship channel	8.8	22.2	21.9	0.3
66	6/26/2014	13:02 PM	Sacramento River deepwater ship channel	8.8	22.2	21.9	0.3
66	6/26/2014	13:24 PM	Sacramento River deepwater ship channel	9.7	22.3	21.8	0.5
66	6/26/2014	13:45 PM	Sacramento River deepwater ship channel	10.0	22.3	21.8	0.5
66	6/26/2014	14:25 PM	Sacramento River deepwater ship channel	10.8	21.9	21.7	0.2
66	7/7/2014	14:20 PM	Sacramento River deepwater ship channel	9.8	23.3	22.8	0.5
66	8/21/2014	10:34 AM	Sacramento River deepwater ship channel	8.2	22.3	21.7	0.6
66	8/25/2014	10:11 AM	Sacramento River deepwater ship channel	9.1	21.4	21.4	0.0
66	10/21/2014	11:11 AM	Sacramento River deepwater ship channel	9.9	19.3	19.2	0.1
66	10/23/2014	10:42 AM	Sacramento River deepwater ship channel	9.5	19.2	19.1	0.1
70	8/25/2014	10:47 AM	Sacramento River deepwater ship channel	10.1	22.1	22.0	0.1
70	10/21/2014	11:48 AM	Sacramento River deepwater ship channel	10.5	19.8	19.6	0.2
70	10/23/2014	11:06 AM	Sacramento River deepwater ship channel	10.4	19.5	19.4	0.1
74	7/7/2014	10:57 AM	Sacramento River deepwater ship channel	11.4	24.0	23.4	0.6
74	7/7/2014	11:01 AM	Sacramento River deepwater ship channel	11.4	24.0	23.4	0.6
74	7/7/2014	11:17 AM	Sacramento River deepwater ship channel	11.4	23.9	23.4	0.5
74	7/7/2014	11:32 AM	Sacramento River deepwater ship channel	11.5	23.9	23.4	0.5

74	7/7/2014	11:50 AM	Sacramento River deepwater ship channel	10.8	23.8	23.4	0.4
74	7/7/2014	12:04 PM	Sacramento River deepwater ship channel	11.5	24.5	23.4	1.1
74	7/7/2014	12:17 PM	Sacramento River deepwater ship channel	11.5	24.2	23.4	0.8
74	7/7/2014	12:31 PM	Sacramento River deepwater ship channel	11.6	24.5	23.4	1.1
74	7/7/2014	12:45 PM	Sacramento River deepwater ship channel	10.5	24.4	23.4	1.1
74	7/7/2014	13:00 PM	Sacramento River deepwater ship channel	11.4	24.5	23.4	1.1
74	7/7/2014	13:17 PM	Sacramento River deepwater ship channel	11.4	24.2	23.4	0.8
74	7/7/2014	13:31 PM	Sacramento River deepwater ship channel	10.8	24.3	23.4	0.9
74	8/21/2014	11:36 AM	Sacramento River deepwater ship channel	10.0	23.0	22.6	0.4
74	8/25/2014	11:19 AM	Sacramento River deepwater ship channel	10.1	22.6	22.4	0.2
74	8/25/2014	1:25 PM	Sacramento River deepwater ship channel	10.2	23.2	22.4	0.8
74	10/21/2014	12:39 PM	Sacramento River deepwater ship channel	11.4	20.0	19.8	0.2
74	10/23/2014	11:27 AM	Sacramento River deepwater ship channel	9.7	19.8	19.6	0.2
76	8/25/2014	11:48 AM	Sacramento River deepwater ship channel	10.4	23.5	23.1	0.4
76	10/21/2014	13:09 PM	Sacramento River deepwater ship channel	10.9	20.4	20.0	0.4
76	10/23/2014	11:52 AM	Sacramento River deepwater ship channel	11.1	20.3	19.9	0.4
84	8/21/2014	12:12 PM	Sacramento River deepwater ship channel	10.8	24.8	23.8	1.0
84	8/25/2014	12:16 PM	Sacramento River deepwater ship channel	9.6	24.2	23.7	0.5
84	8/25/2014	1:03 PM	Sacramento River deepwater ship channel	10.1	24.4	23.7	0.7
84	10/21/2014	13:42 PM	Sacramento River deepwater ship channel	11.4	21.0	20.5	0.5
84	10/23/2014	12:17 PM	Sacramento River deepwater ship channel	11.0	20.7	20.1	0.6

Fig 1. Sites where vertical profiles of water temperature were collected, April-October 2014.

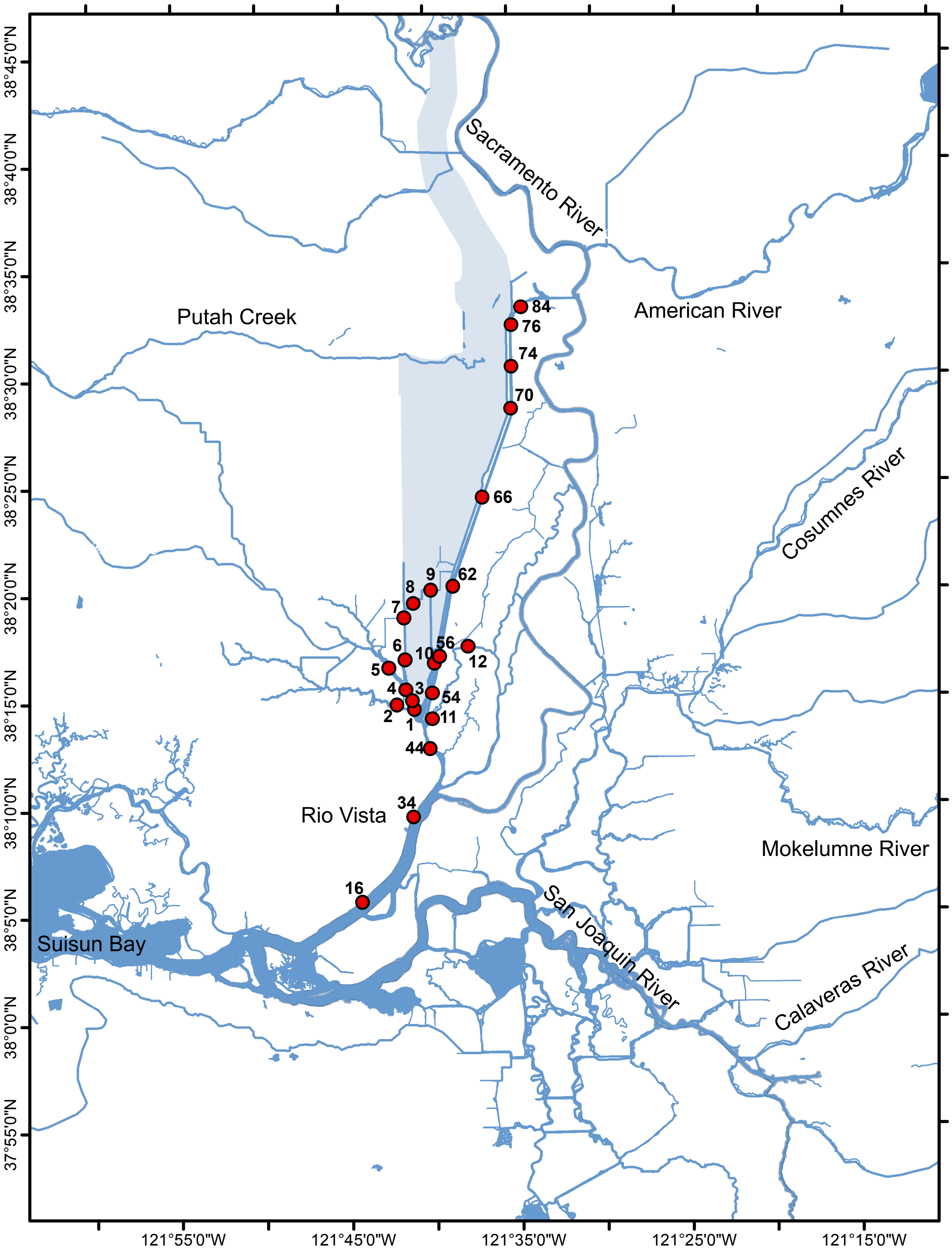

Sacramento River
American River
Putah Creek
Cosumnes River
Mokelumne River
San Joaquin River
Calaveras River
Rio Vista
Suisun Bay
84
76
74
70
66
62
9
8
7
6
5
56
10
12
4
3
54
2
1
11
44
34
16
38°45'0"N
38°40'0"N
38°35'0"N
38°30'0"N
38°25'0"N
38°20'0"N
38°15'0"N
38°10'0"N
38°5'0"N
38°0'0"N
37°55'0"N
121°55'0"W
121°45'0"W
121°35'0"W
121°25'0"W
121°15'0"W
