# Supplementary material for: Coupled Downscaled Climate Models and Ecophysiological Metrics Forecast Habitat Compression for an Endangered Estuarine Fish
Source: PLoS One. 2016 Jan 21;11(1):e0146724. doi: 10.1371/journal.pone.0146724 (PMC4721863; doi:10.1371/journal.pone.0146724)
Supplement: S2 Table — (PDF) [file pone.0146724.s007.pdf]

S2 Table. Median, minimum, and maximum values for the number of days per year when mean daily water temperature is ≥24°C, during each decade from 2010-2099, for the juvenile life stage of Delta Smelt (June-December) for the least-warming (PCM-B1), most-warming (GFDL-A2) and two intermediate (PCM-A2 and GFDL-B1) climate change scenarios. The significance value for Trend is from the Mann-Kendal test (NS, P≥0.05; *, P<0.05; **, P<0.01; ***, P<0.001; NA, no non-zero values; NT, fewer than 3 values so trend not calculated) and the number is the slope of a regression of decadal medians.

	2010-2019			2020-2029			2030-2039			2040-2049			2050-2059			2060-2069			2070-2079			2080-2089			2090-2099			
	Median	Mini-mum	Maxi-mum	Median	Mini-mum	Maxi-mum	Median	Mini-mum	Maxi-mum	Median	Mini-mum	Maxi-mum	Median	Mini-mum	Maxi-mum	Median	Mini-mum	Maxi-mum	Median	Mini-mum	Maxi-mum	Median	Mini-mum	Maxi-mum	Median	Mini-mum	Maxi-mum	Trend
**Scenario GFDL-A2**																												
**San Joaquin River**																												
Mossdale	93	70	118	93.5	77	121	107	72	118	103.5	87	129	120	96	124	122.5	110	135	126	108	137	130.5	117	142	136.5	133	151	5.7***
Burns Cut	112	104	130	119	87	130	120	78	124	118	102	138	130	117	134	133.5	116	143	132.5	115	148	139.5	128	145	144.5	137	152	3.87**
Prisoners Point	66.5	35	98	68	63	104	82.5	57	93	75	62	117	101.5	67	121	102.5	87	122	105	97	136	129.5	102	136	130	111	145	9.38***
Jersey Point	69.5	23	104	70.5	57	113	79.5	60	98	81	67	119	109	72	124	109.5	100	126	114.5	102	129	134.5	103	144	137.5	125	152	9.38***
Antioch	57	21	93	63.5	55	100	74.5	49	88	68.5	56	115	100	58	122	0.5	90	122	107	98	137	28.5	97	137	133	117	151	9.93***
**Sacramento River**																												
Hood	26.5	2	56	43	18	86	45.5	27	76	48	32	78	66.5	41	98	75	57	110	95	70	121	108	74	132	123.5	94	137	11.82***
Rio Vista	40.5	18	62	48.5	33	90	55.5	38	82	56.5	44	92	84.5	51	114	90	72	117	101	88	130	117	93	136	128	103	147	11.33***
Decker Island	42	5	67	46.5	18	94	64	39	83	63	44	95	89.5	48	111	89	82	112	102.5	91	112	119.5	99	129	133.5	114	146	11.47***
**North Delta**																												
Upper Cache Slough	9	0	22	8.5	0	62	17.5	0	43	26.5	3	46	32	13	77	39	30	74	60.5	44	91	74.5	57	110	116.5	86	127	12.11***
Miners Slough	13.5	0	28	17	0	66	24	3	57	29.5	9	50	40	23	80	44.5	32	87	67.5	52	94	86	59	113	118	87	127	12.12***
Liberty Island	36	8	61	42.5	25	88	54	37	78	54	43	93	87	49	113	85	69	117	100	80	120	116.5	94	131	132	107	148	12.04***
Deepwater Ship Channel	18.5	0	50	27	4	76	33	19	70	37.5	16	61	50.5	30	86	55	52	102	82.5	58	110	98.5	65	118	123.5	91	132	12.52***
Lower Cache Slough	15.5	0	38	21.5	1	73	27.5	12	64	32	11	56	44.5	29	84	49.5	37	93	77	57	106	94.5	60	115	122.5	88	133	12.73***
**Confluence**																												
Mallard Island	17.5	0	48	35	9	82	29.5	16	71	39	13	66	51	30	84	63	43	96	86	62	119	100	61	133	121.5	90	135	12.47***
**Suisun Bay**																												
Martinez	0	0	13	0	0	40	10	0	23	21.5	0	36	15	0	64	35	22	61	45	21	83	68	43	104	102.5	77	116	11.63***
**Scenario GFDL-B1**																												
**San Joaquin River**																												
Mossdale	84.5	77	118	95	84	112	99	75	112	104.5	84	119	101	76	123	111	87	139	111.5	88	120	116	97	132	109	84	122	3.21**
Burns Cut	107	92	134	112.5	94	122	111	87	127	114.5	108	131	112.5	88	132	125	110	146	123	106	130	128	111	139	127.5	101	139	2.72**
Prisoners Point	61	39	86	69.5	52	84	74	48	90	79	59	101	73.5	49	100	92.5	62	109	94.5	73	114	102	82	116	94	60	110	4.73**
Jersey Point	64.5	40	100	72.5	58	97	73.5	26	100	80.5	53	102	79.5	54	103	93.5	62	116	98.5	59	115	112	83	123	91.5	65	109	4.83**
Antioch	57	37	86	56	40	69	67.5	18	92	72	49	98	69.5	46	99	84	52	106	89	55	114	100	73	118	91.5	58	112	5.42**
**Sacramento River**																												
Hood	29.5	12	65	25.5	0	41	47	3	81	45.5	33	89	46	13	83	56	18	79	61	27	92	63	30	104	66	54	91	4.95**
Rio Vista	38	21	75	39	4	50	56.5	8	85	57	44	94	59	33	90	70.5	36	87	70	39	107	79	48	114	79.5	56	101	5.44***
Decker Island	36	13	54	45	23	87	57	0	92	60.5	44	93	55.5	29	95	72.5	22	100	81	39	109	96	55	110	80	55	95	6.48**
**North Delta**																												
Upper Cache Slough	6	1	24	8.5	0	45	20	0	62	17	13	55	20	0	43	33	0	58	32.5	8	49	29	11	100	31.5	0	76	3.41*
Miners Slough	8.5	2	25	12	0	51	25	0	65	22	14	62	22	0	57	39.5	0	63	42	12	55	36.5	14	100	35.5	0	79	3.88*
Liberty Island	32	13	51	41	18	77	51.5	2	85	53.5	43	95	51.5	30	96	65.5	26	86	71	37	101	82.5	47	114	75	51	95	5.49***
Deepwater Ship Channel	18.5	6	33	21	0	62	35	0	78	34	24	80	30.5	8	76	46.5	8	74	49.5	19	70	54.5	24	102	50	8	88	4.47**
Lower Cache Slough	11	5	28	12.5	0	54	30.5	0	72	27	19	75	23.5	3	70	42.5	0	67	46	15	63	45	19	102	42.5	2	81	4.5*
**Confluence**																												
Mallard Island	17	6	59	14.5	0	40	38	0	75	29.5	24	82	31.5	5	77	49	1	74	48.5	20	79	46.5	7	103	49.5	41	85	4.44**
**Suisun Bay**																												
Martinez	0	0	21	0	0	30	8	0	52	7.5	0	44	2	0	20	24	0	56	8	0	40	10	0	93	25	3	67	2.44**
**Scenario PCM-A2**																												
**San Joaquin River**																												
Mossdale	87.5	68	107	92.5	85	100	94.5	83	116	108	82	126	108.5	80	124	112.5	86	122	111	94	130	117	113	136	134	109	150	4.95***

Burns Cut	104.5	91	122	104	91	124	112	100	139	117.5	94	147	124.5	107	137	124.5	104	136	121.5	112	143	131	121	149	138.5	115	154	4.05**
Prisoners Point	58	28	79	74	49	82	63.5	44	92	84.5	53	94	81.5	63	113	83.5	65	105	96.5	76	120	104	94	118	108.5	96	138	5.95**
Jersey Point	56	25	86	68.5	50	81	65.5	35	92	88.5	65	103	79.5	55	118	86.5	75	109	97.5	66	124	107.5	94	116	120	98	140	7.25**
Antioch	46.5	16	70	66	40	75	56.5	30	91	80.5	52	91	73.5	51	115	76	57	100	93.5	64	117	103	88	118	111	97	138	7.31**
**Sacramento River**																												
Hood	11	0	40	27.5	14	44	39.5	13	49	43	28	71	52	26	85	50	11	76	67	49	107	75	63	96	84.5	65	130	8.31***
Rio Vista	21.5	5	63	45	28	63	48.5	20	70	64.5	48	78	64.5	36	104	69	39	89	81	59	112	87	68	113	96.5	81	135	8.26***
Decker Island	15	0	65	45.5	30	61	47	19	69	69.5	42	78	64.5	36	107	71	58	93	84.5	61	116	94	75	100	103.5	93	136	9.6***
**North Delta**																												
Upper Cache Slough	0	0	16	6	0	19	6	0	17	16	1	32	20.5	0	49	27	1	65	39	29	73	42.5	34	62	58	41	93	6.98***
Miners Slough	0.5	0	21	10	0	25	14.5	0	20	22	8	37	29.5	0	48	35	1	69	46.5	33	76	53	41	67	67	56	102	7.87***
Liberty Island	17.5	4	58	39	26	58	47	17	61	61	38	77	59.5	33	106	66.5	43	87	83.5	60	115	90	75	101	101	87	136	9.42***
Deepwater Ship Channel	6	0	29	20.5	6	29	28.5	11	38	36.5	18	49	41	3	70	42.5	33	73	60	40	90	60.5	54	77	87.5	69	120	8.58***
Lower Cache Slough	1.5	0	21	15	0	26	20.5	3	29	26.5	9	39	34	1	67	38.5	14	71	55	40	84	55.5	47	72	80	64	117	8.61***
**Confluence**																												
Mallard Island	2	0	26	17	4	27	27.5	1	37	32	15	41	42	18	72	39	0	72	57	42	92	61	57	88	79.5	59	128	8.47***
**Suisun Bay**																												
Martinez	0	0	6	0	0	7	0	0	0	2.5	0	17	10	0	25	3	0	45	22.5	0	52	36	23	49	44.5	15	74	5.53***
**Scenario PCM-B1**																												
**San Joaquin River**																												
Mossdale	81.5	50	103	82	64	93	86.5	68	106	78.5	61	97	87.5	69	123	90.5	70	104	90.5	74	121	109.5	61	119	90.5	86	111	2.31**
Burns Cut	93	87	112	100	85	112	104	80	116	96.5	85	120	108.5	92	132	110.5	85	125	108	96	141	118	80	129	112	95	135	2.53**
Prisoners Point	36.5	8	69	44.5	20	69	51	34	86	47.5	32	80	59.5	43	81	59	45	78	67.5	47	107	83.5	34	94	63	42	89	4.46**
Jersey Point	61.5	13	79	56.5	36	83	69	54	91	65.5	54	86	75.5	54	121	80.5	61	88	85	52	116	96	50	117	81.5	68	99	4.09**
Antioch	29.5	0	65	37.5	7	57	35.5	13	69	37.5	20	78	48	36	82	53.5	39	66	64	36	109	74	28	100	54	39	81	4.68**
**Sacramento River**																												
Hood	7.5	0	55	10	0	41	6.5	0	43	12.5	0	29	24	6	47	20.5	3	39	36	0	59	33	8	72	34	9	63	4.03**
Rio Vista	14	0	58	22.5	1	47	18.5	0	50	28.5	3	49	31.5	14	63	40.5	24	49	54	12	67	57.5	22	81	45	23	70	5.2**
Decker Island	29	0	63	42	6	63	41.5	23	71	44	17	74	55.5	39	92	61	44	73	67	32	105	81	31	103	67	50	84	5.62***
**North Delta**																												
Upper Cache Slough	0	0	13	0	0	29	0	0	37	4.5	0	21	11.5	1	34	17	0	28	20	0	41	24.5	0	59	18	0	40	3.3**
Miners Slough	2.5	0	24	4	0	37	2.5	0	39	10	0	24	18.5	1	41	20.5	2	37	30.5	0	52	29.5	3	70	29.5	7	51	4.18**
Liberty Island	28	0	46	37	8	56	37.5	13	68	38.5	25	77	48	36	83	55.5	47	66	63.5	29	111	77	36	108	53	45	80	4.94**
Deepwater Ship Channel	11.5	0	32	17	0	44	14.5	0	46	24.5	7	39	26.5	10	48	32	20	42	47	7	62	45	22	75	43	17	65	4.71**
Lower Cache Slough	6.5	0	28	10	0	39	7.5	0	43	17.5	0	32	25	4	44	28	7	38	39	1	58	35.5	6	73	34	9	62	4.33**
**Confluence**																												
Mallard Island	0	0	36	0	0	29	0	0	34	0	0	20	9	0	36	10	0	29	21	0	42	22	0	59	18.5	0	44	3.2**
**Suisun Bay**																												
Martinez	0	0	9	0	0	0	0	0	7	0	0	0	0	0	1	0	0	0	0	0	0	0	0	19	0	0	14	NA
