# Supplementary material for: Coupled Downscaled Climate Models and Ecophysiological Metrics Forecast Habitat Compression for an Endangered Estuarine Fish
Source: PLoS One. 2016 Jan 21;11(1):e0146724. doi: 10.1371/journal.pone.0146724 (PMC4721863; doi:10.1371/journal.pone.0146724)
Supplement: S3 Table — (PDF) [file pone.0146724.s008.pdf]

S3 Table. Median, minimum, and maximum values for the number of days per year for the duration of the spawning window (15-20°C), during each decade from 2010-2099, for the adult life stage of Delta Smelt for the least-warming (PCM-B1), most-warming (GFDL-A2) and two intermediate (PCM-A2 and GFDL-B1) climate change scenarios. The significance value for Trend is from the Mann-Kendal test (NS, P≥0.05; *, P<0.05; **, P<0.01; ***, P<0.001; NA, no non-zero values; NT, fewer than 3 values so trend not calculated) and the number is the slope of a regression of decadal medians.

	2010-2019			2020-2029			2030-2039			2040-2049			2050-2059			2060-2069			2070-2079			2080-2089			2090-2099			
	Median	Mini-mum	Maxi-mum	Median	Mini-mum	Maxi-mum	Median	Mini-mum	Maxi-mum	Median	Mini-mum	Maxi-mum	Median	Mini-mum	Maxi-mum	Median	Mini-mum	Maxi-mum	Median	Mini-mum	Maxi-mum	Median	Mini-mum	Maxi-mum	Median	Mini-mum	Maxi-mum	Trend
**Scenario GFDL-A2**																												
**San Joaquin River**																												
Mossdale	52.0	17	75	53.5	20	79	49.5	21	58	53.5	28	71	50.5	43	74	45.5	36	80	50.0	30	62	63.5	22	81	45.0	33	69	NS
Burns Cut	47.5	32	66	47.5	20	58	45.5	20	51	47.0	26	58	49.0	43	69	42.0	35	73	49.0	28	60	55.0	31	64	41.5	25	59	NS
Prisoners Point	54.0	33	79	52.5	19	82	50.0	25	58	55.5	28	64	58.5	46	77	53.5	43	84	59.0	35	73	61.5	38	78	48.5	27	76	NS
Jersey Point	47.5	28	72	46.0	23	66	46.5	15	58	49.5	40	60	53.5	29	79	55.5	42	89	61.5	32	79	56.5	30	84	52.5	19	76	1.46*
Antioch	53.5	29	80	54.0	22	87	50.0	21	58	55.5	41	65	58.0	51	79	57.0	44	87	63.0	32	79	69.0	31	84	54.5	20	80	1.28*
**Sacramento River**																												
Hood	42.0	28	68	44.5	23	67	39.0	20	59	51.0	36	68	49.0	27	71	46.0	39	66	51.5	32	73	54.0	39	74	52.5	19	74	1.51**
Rio Vista	42.5	28	70	45.0	22	66	39.5	20	58	51.5	36	68	49.5	27	71	46.0	39	66	58.0	33	68	53.0	40	85	56.5	18	75	1.86*
Decker Island	48.0	33	70	51.5	22	71	41.0	20	80	53.5	37	77	53.0	28	76	52.5	41	88	69.0	33	81	54.5	40	86	57.0	20	75	1.67*
**North Delta**																												
Upper Cache Slough	50.0	27	72	45.5	22	72	42.0	17	58	53.0	38	70	58.0	27	81	55.0	42	98	63.5	32	82	72.0	43	96	46.5	17	85	NS
Miners Slough	42.5	28	71	47.5	22	69	45.0	18	58	54.0	40	70	57.0	27	81	58.0	41	89	61.5	32	80	67.0	41	88	58.0	18	79	2.63**
Liberty Island	43.0	27	71	46.5	23	67	44.0	11	65	51.0	36	70	58.0	26	80	54.5	39	89	58.5	32	76	63.0	39	87	62.5	17	77	2.85*
Deepwater Ship Channel	52.0	27	82	46.5	16	66	44.0	18	58	49.0	38	68	58.0	19	81	57.0	37	96	63.0	32	82	74.5	30	96	44.0	18	83	NS
Lower Cache Slough	50.0	28	72	46.0	21	71	45.0	18	58	53.0	38	78	58.0	26	80	58.5	42	97	64.0	32	82	74.0	42	96	44.5	17	82	NS
**Confluence**																												
Mallard Island	51.5	32	73	54.0	23	71	46.5	21	83	54.5	38	78	58.5	30	78	58.5	43	90	71.0	35	83	56.5	42	87	63.0	20	76	1.78*
**Suisun Bay**																												
Martinez	59.0	38	90	63.0	24	80	51.5	22	94	61.0	38	79	60.5	30	81	60.0	46	90	72.5	35	102	58.0	44	98	62.0	22	79	NS
**Scenario GFDL-B1**																												
**San Joaquin River**																												
Mossdale	44.5	21	62	35.0	19	71	60.5	29	81	53.0	18	70	38.0	23	96	46.0	34	89	52.0	27	95	47.5	27	69	53.5	17	86	NS
Burns Cut	43.5	22	57	34.0	24	59	49.5	26	65	49.5	21	62	43.0	27	57	46.5	39	64	41.0	27	57	46.0	28	60	40.5	18	73	NS
Prisoners Point	50.5	23	60	41.5	30	71	59.5	27	81	54.5	38	71	47.5	36	76	51.0	38	78	51.0	29	81	55.0	33	80	46.5	20	83	NS
Jersey Point	51.5	27	58	41.0	30	63	55.5	27	88	55.0	38	74	49.5	36	82	47.5	31	87	55.5	33	98	56.0	31	65	52.5	29	79	NS
Antioch	48.5	29	65	39.0	30	64	60.5	27	105	53.5	39	75	49.0	36	80	52.5	38	82	50.5	33	88	56.5	40	66	58.0	20	84	NS
**Sacramento River**																												
Hood	50.0	26	56	43.0	28	61	50.5	34	86	48.0	15	64	45.5	28	82	52.0	23	86	56.0	32	93	45.0	31	57	51.5	34	72	NS
Rio Vista	49.5	26	55	41.0	28	61	50.5	37	87	51.0	15	65	46.0	28	82	51.5	23	86	56.5	32	93	44.5	31	56	50.5	33	69	NS
Decker Island	53.0	19	70	49.0	31	61	63.5	33	89	55.5	19	70	47.5	29	85	55.0	31	87	59.5	33	96	47.0	31	66	52.0	34	72	NS
**North Delta**																												
Upper Cache Slough	52.0	27	68	50.5	31	73	64.0	25	113	56.5	36	85	47.5	17	83	50.5	30	117	55.5	31	103	55.5	30	83	63.5	20	83	NS
Miners Slough	52.0	27	61	46.5	30	63	59.0	25	98	56.0	36	74	51.5	34	83	50.0	31	88	55.5	31	101	53.5	31	66	59.0	20	78	NS
Liberty Island	52.5	27	58	44.5	31	62	57.5	25	88	56.0	38	74	47.5	34	83	51.5	31	88	58.0	32	102	53.5	30	66	53.0	20	74	NS
Deepwater Ship Channel	49.0	29	60	40.0	31	72	64.5	26	112	55.5	37	83	46.0	20	84	49.5	33	116	50.0	32	102	56.5	40	81	60.0	20	79	NS
Lower Cache Slough	53.0	28	68	47.0	31	64	64.0	25	112	57.0	36	83	47.5	21	83	51.0	34	116	56.5	32	102	56.5	30	82	60.5	20	81	NS
**Confluence**																												
Mallard Island	55.0	37	70	50.0	35	63	64.0	39	89	63.5	42	74	49.0	30	85	59.0	31	88	65.5	33	101	51.0	32	69	61.0	36	82	NS
**Suisun Bay**																												
Martinez	58.5	43	73	56.0	39	70	73.0	40	103	63.0	21	75	53.0	47	87	62.0	39	95	65.5	40	98	53.0	33	73	63.5	38	82	NS

														**Scenario PCM-A2**														
**San Joaquin River**																												
Mossdale	48.0	28	65	52.0	40	69	51.5	20	72	49.0	33	55	52.5	34	77	58.5	15	75	40.0	34	63	50.5	34	78	51.0	28	77	NS
Burns Cut	43.0	28	62	49.0	40	55	45.0	30	69	43.5	26	49	42.5	30	63	50.0	20	64	39.0	32	56	50.0	30	66	39.0	28	72	NS
Prisoners Point	56.5	35	66	55.5	42	77	51.0	37	77	47.5	25	58	50.5	32	68	65.0	20	73	50.0	39	66	55.5	44	68	47.0	29	77	NS
Jersey Point	53.5	23	77	50.5	33	88	59.5	37	79	43.0	27	60	52.0	31	82	61.0	24	82	57.0	42	80	53.0	34	75	42.0	31	69	NS
Antioch	51.5	30	69	54.5	42	87	57.5	37	79	45.0	22	72	51.0	32	71	69.0	21	77	57.5	43	68	62.5	43	74	46.0	33	79	NS
**Sacramento River**																												
Hood	52.5	22	69	49.5	32	88	61.5	48	74	47.5	27	65	53.0	31	80	62.5	49	85	57.5	47	76	51.0	35	70	41.5	27	65	NS
Rio Vista	52.5	22	77	49.5	32	88	58.5	37	74	42.0	27	60	53.5	31	80	62.0	49	82	57.5	44	78	50.5	33	70	41.5	27	65	NS
Decker Island	54.5	24	66	62.5	33	80	60.0	45	77	48.0	26	70	51.5	34	83	65.5	49	87	59.5	47	76	53.5	41	71	43.5	27	65	NS
**North Delta**																												
Upper Cache Slough	54.0	14	88	52.0	32	91	60.5	39	81	42.5	17	84	58.5	30	117	64.5	20	92	58.5	30	83	53.0	32	93	42.5	25	68	NS
Miners Slough	54.0	15	84	54.0	32	90	60.0	39	80	43.0	17	82	59.0	31	116	64.0	19	91	60.0	46	83	53.0	33	77	42.5	27	67	NS
Liberty Island	53.5	14	83	54.0	31	90	59.5	38	81	42.5	26	72	58.5	31	82	63.5	19	90	58.5	46	82	55.0	32	76	42.0	16	68	NS
Deepwater Ship Channel	53.0	12	75	50.0	32	90	58.0	37	80	42.0	17	76	50.5	30	88	66.0	20	93	59.0	38	82	60.0	33	89	43.5	23	71	NS
Lower Cache Slough	54.0	14	85	50.5	32	90	62.0	38	80	43.0	18	83	58.0	30	117	66.5	19	92	60.0	31	83	53.5	33	87	42.5	32	69	NS
**Confluence**																												
Mallard Island	55.0	25	82	64.5	33	90	63.0	49	82	49.0	27	73	55.0	39	83	67.0	51	90	61.0	49	81	57.0	41	77	44.0	28	68	NS
**Suisun Bay**																												
Martinez	58.0	41	84	71.0	34	94	68.5	58	79	58.5	29	85	78.5	45	86	69.5	56	93	63.0	49	84	71.5	42	85	58.0	32	69	NS
														**Scenario PCM-B1**														
**San Joaquin River**																												
Mossdale	46.0	29	76	47.5	9	65	36.0	11	66	52.5	26	78	45.5	20	59	29.0	17	48	44.0	26	60	48.0	36	59	53.0	27	66	NS
Burns Cut	43.0	33	68	44.5	27	60	35.5	29	55	46.0	30	69	44.0	32	54	34.5	28	59	43.5	29	57	44.5	32	56	49.0	27	57	NS
Prisoners Point	53.5	41	74	51.0	43	68	38.0	32	64	49.0	36	75	51.0	32	72	47.5	26	68	54.0	38	79	55.0	32	91	52.0	31	72	NS
Jersey Point	56.5	36	65	52.0	36	70	50.5	30	88	54.5	34	78	55.0	39	65	58.0	32	71	55.0	33	75	52.0	28	93	56.5	30	78	NS
Antioch	57.5	42	72	55.5	42	70	44.5	31	65	53.5	38	75	56.0	26	72	57.0	33	74	60.0	37	77	53.0	32	93	54.5	32	80	NS
**Sacramento River**																												
Hood	57.0	21	64	47.0	35	70	50.5	25	87	49.5	34	79	52.0	35	63	57.5	30	71	53.5	35	75	51.0	19	84	53.5	30	78	NS
Rio Vista	57.0	21	64	47.0	35	70	52.0	29	87	49.5	34	79	53.5	35	64	57.0	30	71	51.0	36	75	51.5	19	91	54.5	30	77	NS
Decker Island	57.5	25	66	51.5	36	72	63.0	31	88	50.0	35	90	55.5	37	71	55.0	29	72	57.0	38	76	51.0	21	85	55.5	31	85	NS
**North Delta**																												
Upper Cache Slough	55.0	26	64	58.5	33	73	48.0	32	68	49.5	33	83	56.0	16	66	59.5	20	82	58.5	33	78	52.5	17	93	58.0	28	86	NS
Miners Slough	57.0	34	65	53.5	34	72	51.0	30	68	49.5	34	82	55.5	18	65	58.5	20	78	57.0	33	76	52.0	18	94	57.5	29	86	NS
Liberty Island	57.5	27	65	53.5	34	72	48.5	29	67	50.0	34	82	54.5	37	76	58.0	31	78	56.5	33	77	52.0	17	93	53.5	30	85	NS
Deepwater Ship Channel	57.0	40	65	57.0	38	71	46.0	31	65	53.5	38	81	55.5	23	65	58.5	21	74	62.0	34	78	52.5	29	94	57.5	32	81	NS
Lower Cache Slough	56.0	34	65	57.5	34	72	47.5	30	65	57.0	34	82	56.5	17	65	59.5	20	82	57.0	33	78	52.5	31	93	58.0	33	86	NS
**Confluence**																												
Mallard Island	58.5	40	66	58.5	36	74	63.0	31	89	57.0	37	83	60.5	38	75	59.5	31	78	58.5	45	77	53.0	21	94	60.5	32	85	NS
**Suisun Bay**																												
Martinez	59.5	26	72	60.5	38	79	72.0	34	90	64.5	52	93	60.0	39	77	60.0	32	81	61.0	47	79	58.5	37	95	66.5	35	89	NS
