# Supplementary material for: Coupled Downscaled Climate Models and Ecophysiological Metrics Forecast Habitat Compression for an Endangered Estuarine Fish
Source: PLoS One. 2016 Jan 21;11(1):e0146724. doi: 10.1371/journal.pone.0146724 (PMC4721863; doi:10.1371/journal.pone.0146724)
Supplement: S4 Table — (PDF) [file pone.0146724.s009.pdf]

S4 Table. Median, minimum, and maximum values for the julian date of the beginning of the spawning window (15-20°C) each year, during each decade from 2010-2099, for the adult life stage of Delta Smelt for the least-warming (PCM-B1), most-warming (GFDL-A2) and two intermediate (PCM-A2 and GFDL-B1) climate change scenarios. The significance value for Trend is from the Mann-Kendal test (NS, P≥0.05; *, P<0.05; **, P<0.01; ***, P<0.001; NA, no non-zero values; NT, fewer than 3 values so trend not calculated) and the number is the slope of a regression of decadal medians.

	2010-2019			2020-2029			2030-2039			2040-2049			2050-2059			2060-2069			2070-2079			2080-2089			2090-2099			
	Median	Minimum	Maximum	Median	Minimum	Maximum	Median	Minimum	Maximum	Median	Minimum	Maximum	Median	Minimum	Maximum	Median	Minimum	Maximum	Median	Minimum	Maximum	Median	Minimum	Maximum	Median	Minimum	Maximum	Trend
**Scenario GFDL-A2**																												
**San Joaquin River**																												
Mossdale	78.5	60	97	75.5	55	102	81.0	69	95	75.5	52	101	72.5	59	94	75.0	42	87	68.5	56	77	49.0	34	78	57.5	51	76	-3.56**
Burns Cut	84.0	77	98	82.0	73	104	88.0	81	97	81.0	73	103	77.0	68	95	78.5	52	88	70.5	64	78	68.5	54	88	65.0	59	87	-2.57**
Prisoners Point	82.5	75	99	84.5	70	105	86.5	81	96	80.0	73	102	75.5	67	95	77.5	51	88	70.0	62	78	64.0	48	79	64.5	57	87	-2.82**
Jersey Point	93.0	79	112	96.5	78	109	98.5	85	119	93.0	81	107	83.5	69	105	83.0	51	93	72.5	65	104	75.5	55	91	70.5	61	98	-3.58**
Antioch	89.5	77	105	86.0	72	108	91.0	82	103	84.5	79	105	77.0	67	96	79.0	50	90	70.0	63	85	64.0	48	87	64.5	52	96	-3.56**
**Sacramento River**																												
Hood	98.0	82	113	101.0	92	110	103.0	88	120	98.5	81	112	93.0	77	108	93.5	75	100	81.0	72	105	82.5	69	92	79.5	64	99	-2.98**
Rio Vista	97.0	81	113	100.5	92	110	103.0	88	120	97.5	81	112	93.0	76	108	91.0	68	100	78.5	72	105	82.5	57	92	78.5	63	99	-3.06**
Decker Island	99.0	82	114	101.5	92	111	104.5	89	124	99.0	82	113	93.5	77	109	90.0	54	102	79.5	69	105	79.0	57	92	79.0	63	99	-3.44**
**North Delta**																												
Upper Cache Slough	94.5	76	112	96.5	67	109	95.0	84	119	93.0	79	105	79.0	66	107	83.5	42	99	70.5	62	87	56.5	46	88	67.5	52	98	-4.78**
Miners Slough	95.5	78	112	98.0	70	109	95.0	85	119	93.0	80	105	86.5	67	107	83.5	51	94	72.5	64	104	69.0	47	89	69.5	58	98	-4.09***
Liberty Island	96.5	79	113	100.5	78	109	99.0	86	120	96.5	80	112	87.5	68	108	84.5	51	99	74.5	65	105	77.5	48	91	71.0	61	99	-4.92**
Deepwater Ship Channel	90.5	76	112	95.5	67	108	92.5	84	119	91.5	79	104	79.5	66	106	79.5	43	93	70.0	62	86	54.5	39	88	64.5	53	97	-4.73**
Lower Cache Slough	92.0	76	112	96.5	67	109	94.5	84	119	91.5	79	105	79.5	67	107	82.5	43	93	70.5	62	87	56.0	39	88	65.5	55	98	-4.74**
**Confluence**																												
Mallard Island	95.5	79	113	98.5	78	110	99.5	86	120	96.0	81	112	87.0	75	107	84.0	52	98	76.5	67	104	78.0	56	91	72.5	62	99	-3.53**
**Suisun Bay**																												
Martinez	97.0	80	113	101.0	79	110	103.5	88	124	98.5	81	113	93.0	76	109	86.0	53	100	78.5	68	105	78.5	57	92	75.5	62	99	-3.60**
**Scenario GFDL-B1**																												
**San Joaquin River**																												
Mossdale	91.5	73	98	85.5	77	107	71.0	41	96	74.0	57	93	78.0	67	97	72.0	50	88	69.5	41	99	74.0	54	94	70.0	45	81	-2.09*
Burns Cut	92.5	76	100	93.0	84	108	78.0	65	96	79.5	70	94	85.0	71	98	76.5	70	88	86.5	62	101	78.5	62	95	78.5	61	96	NS
Prisoners Point	93.5	74	99	92.5	80	108	77.0	65	96	79.0	68	95	85.0	70	98	76.0	69	90	86.0	60	100	78.0	56	95	78.0	60	95	NS
Jersey Point	103.0	84	108	101.0	90	113	87.5	66	99	90.5	71	99	96.0	73	113	85.0	77	94	89.0	66	107	82.5	62	104	89.5	66	106	-1.87*
Antioch	96.0	76	106	94.5	87	109	78.5	47	98	82.0	70	98	88.0	71	101	77.0	70	92	87.5	60	105	80.5	60	96	80.0	60	98	NS
**Sacramento River**																												
Hood	103.5	90	111	103.5	91	117	95.0	68	103	96.5	90	113	99.5	87	114	95.5	79	99	95.0	67	108	94.0	66	107	93.0	78	112	-1.19**
Rio Vista	103.5	90	109	103.5	91	117	94.5	67	101	94.5	90	113	99.5	86	114	95.0	79	98	92.5	67	108	94.0	66	106	93.0	76	112	-1.23*
Decker Island	105.5	91	123	105.0	92	118	95.0	67	105	97.0	91	113	100.5	87	115	96.5	79	99	98.0	67	109	95.0	66	109	94.0	76	113	-1.18*
**North Delta**																												
Upper Cache Slough	101.5	74	112	97.0	79	114	88.0	41	100	92.0	68	99	95.5	68	114	77.0	50	95	87.0	59	106	85.5	55	105	81.0	45	99	-2.23**
Miners Slough	102.5	84	108	100.5	89	117	90.5	66	100	92.5	70	99	96.5	71	114	78.5	70	95	88.0	60	107	86.0	59	105	88.0	66	107	-2.01*
Liberty Island	102.5	84	112	103.0	90	117	94.5	66	101	92.5	71	100	99.0	71	114	83.0	77	96	89.0	63	107	87.0	62	106	92.0	75	112	-1.84*
Deepwater Ship Channel	98.5	74	107	93.5	80	112	81.5	42	98	90.5	67	98	92.5	68	113	76.5	51	93	87.0	59	105	80.0	56	96	80.5	65	98	-1.93*
Lower Cache Slough	99.0	74	108	100.5	88	117	82.0	42	99	91.5	68	99	94.0	70	114	77.0	51	94	87.0	59	106	80.5	56	105	80.5	66	99	-2.31*
**Confluence**																												
Mallard Island	103.0	85	109	102.0	90	117	92.0	66	100	92.5	72	100	98.5	75	114	86.0	78	96	89.5	66	108	93.0	63	105	90.5	75	107	NS
**Suisun Bay**																												
Martinez	104.5	90	123	103.5	91	117	94.5	66	103	96.0	83	113	99.5	84	114	90.0	78	98	92.5	66	108	94.0	63	107	93.0	75	113	-1.41*
**Scenario PCM-A2**																												
**San Joaquin River**																												

Mossdale	86.5	77	104	81.5	66	87	85.5	74	92	77.5	68	92	69.5	48	90	70.0	62	89	75.0	67	101	68.0	47	78	70.5	43	85	-2.22*
Burns Cut	92.0	82	105	86.0	73	89	89.5	76	95	86.5	76	93	81.0	74	100	79.0	65	91	79.0	69	99	73.0	56	85	79.5	69	88	-1.96**
Prisoners Point	88.0	80	105	85.0	69	88	88.5	76	95	87.0	74	95	80.0	69	100	77.0	63	91	78.0	69	101	73.0	54	85	75.5	55	86	-1.95**
Jersey Point	102.5	86	118	90.5	75	103	91.0	78	99	92.5	85	109	87.0	76	104	82.5	76	95	84.5	77	104	84.0	56	100	90.5	76	93	NS
Antioch	96.5	82	108	87.0	70	91	89.5	76	97	89.0	76	107	84.0	75	102	79.5	64	92	79.0	69	102	76.5	54	87	79.5	56	91	-2.17**
**Sacramento River**																												
Hood	110.0	93	120	94.5	76	107	92.0	84	101	94.0	88	114	91.0	78	107	84.0	79	95	89.0	79	110	89.5	78	101	93.0	78	103	-1.65*
Rio Vista	110.0	87	120	94.0	76	107	92.0	83	101	94.0	88	114	88.0	78	107	83.5	78	95	89.0	79	109	89.0	78	100	92.5	78	102	NS
Decker Island	111.5	99	121	96.0	76	107	94.5	84	116	94.5	89	117	92.5	78	109	85.0	79	97	89.5	79	111	89.5	72	101	93.0	79	103	-1.88*
**North Delta**																												
Upper Cache Slough	101.0	80	119	89.0	73	103	89.0	76	99	91.5	74	114	86.5	48	107	81.0	63	95	83.0	69	106	88.0	53	100	87.5	50	102	NS
Miners Slough	102.0	82	119	89.5	74	103	89.5	77	99	91.5	76	114	86.5	49	107	82.0	75	95	87.5	77	106	88.0	54	100	88.5	55	102	NS
Liberty Island	109.0	83	120	90.0	75	104	90.5	78	100	92.5	87	116	87.5	76	107	83.0	76	95	87.5	78	109	88.5	54	100	90.5	75	103	NS
Deepwater Ship Channel	101.0	80	118	89.0	68	103	89.0	76	97	90.0	74	108	83.0	48	103	80.0	63	94	80.0	69	103	80.5	53	95	86.5	53	92	NS
Lower Cache Slough	101.0	80	119	89.0	68	103	89.5	77	97	91.5	74	113	86.5	48	103	80.0	63	95	83.0	76	106	87.0	53	100	87.5	53	93	NS
**Confluence**																												
Mallard Island	110.0	86	119	90.5	75	104	91.0	78	100	92.5	86	114	87.5	77	107	83.0	76	95	87.5	78	109	87.5	57	100	91.5	76	102	NS
**Suisun Bay**																												
Martinez	110.5	87	121	91.0	76	104	92.0	83	101	94.0	87	116	88.0	77	108	83.5	78	95	89.0	78	110	89.0	73	101	92.5	77	103	NS
														**Scenario PCM-B1**														
**San Joaquin River**																												
Mossdale	84.5	73	93	84.0	75	111	88.0	65	93	81.5	66	96	85.0	67	93	81.5	73	99	78.0	65	87	74.5	65	89	77.5	57	89	-1.28*
Burns Cut	86.5	82	98	90.0	82	110	90.0	78	95	89.0	75	100	86.0	78	96	85.0	83	101	83.0	68	88	79.5	70	94	79.5	77	91	-1.29**
Prisoners Point	86.0	82	97	90.0	81	111	89.0	69	94	87.0	74	100	86.0	76	96	84.5	82	106	82.5	67	90	78.5	68	95	79.5	77	91	-1.27**
Jersey Point	100.0	86	109	98.0	92	116	97.0	80	108	94.5	77	113	93.5	82	105	92.5	86	110	91.5	75	103	91.5	72	102	86.5	79	98	-1.44***
Antioch	93.0	83	104	93.5	84	113	94.5	69	106	91.5	75	111	87.5	77	101	88.5	84	109	84.5	67	99	82.5	69	97	82.5	77	94	-1.63**
**Sacramento River**																												
Hood	101.5	88	126	100.5	93	118	101.0	82	111	97.0	79	114	99.0	85	109	97.5	87	111	96.5	88	109	95.5	89	110	89.5	80	106	-1.19**
Rio Vista	101.5	88	126	100.5	93	117	100.5	82	109	96.5	79	114	96.5	85	109	97.5	87	111	96.5	88	105	95.0	83	110	89.0	80	105	-1.23**
Decker Island	102.5	89	127	105.0	93	119	101.5	82	112	98.5	79	125	99.5	86	110	101.0	88	112	97.5	89	109	97.0	89	111	91.5	81	106	-1.23**
**North Delta**																												
Upper Cache Slough	100.5	83	117	96.0	84	118	94.0	68	103	93.5	76	113	92.0	76	109	91.0	84	109	92.0	77	103	92.0	67	109	84.5	78	102	-1.38**
Miners Slough	100.5	84	110	97.5	91	117	95.0	80	108	94.5	76	113	92.5	78	109	93.0	85	110	92.0	77	104	92.5	69	109	85.5	78	102	-1.38***
Liberty Island	101.0	85	117	100.0	92	118	97.5	81	109	95.0	77	113	93.5	81	109	96.5	85	110	95.0	77	109	92.5	70	110	87.5	79	106	-1.43***
Deepwater Ship Channel	97.0	83	106	95.5	84	113	94.0	68	102	91.5	76	112	90.0	76	104	89.5	84	109	88.5	67	101	83.5	67	98	84.0	78	96	-1.68***
Lower Cache Slough	100.0	83	110	96.0	84	116	95.0	68	108	94.0	76	113	92.0	77	109	90.0	85	109	91.0	75	103	84.0	67	98	84.5	78	97	-1.83***
**Confluence**																												
Mallard Island	101.0	86	110	100.0	92	117	97.5	81	109	95.0	77	113	93.5	82	109	96.5	86	111	92.5	77	104	92.5	80	109	87.5	79	102	-1.42***
**Suisun Bay**																												
Martinez	101.5	88	127	100.5	93	119	101.0	81	111	96.5	79	125	98.5	85	110	97.5	86	111	96.5	88	109	95.0	81	110	88.5	80	106	-1.28**
