# Supplementary material for: Coupled Downscaled Climate Models and Ecophysiological Metrics Forecast Habitat Compression for an Endangered Estuarine Fish
Source: PLoS One. 2016 Jan 21;11(1):e0146724. doi: 10.1371/journal.pone.0146724 (PMC4721863; doi:10.1371/journal.pone.0146724)
Supplement: S5 Table — (PDF) [file pone.0146724.s010.pdf]

S5 Table. Median, minimum, and maximum values for the julian date of the beginning of the maturation window (last day of 24°C to beginning of the spawning window) each year during each decade from 2010-2099, for the adult life stage of Delta Smelt for the least-warming (PCM-B1), most-warming (GFDL-A2) and two intermediate (PCM-A2 and GFDL-B1) climate change scenarios. The significance value for Trend is from the Mann-Kendal test (NS, P≥0.05; *, P<0.05; **, P<0.01; ***, P<0.001; NA, no non-zero values; NT, fewer than 3 values so trend not calculated) and the number is the slope of a regression of decadal medians.

	2010-2019				2020-2029				2030-2039				2040-2049				2050-2059				2060-2069				2070-2079				2080-2089				2090-2099				
	Sample size	Median	Mini-mum	Maxi-mum	Sample size	Median	Mini-mum	Maxi-mum	Sample size	Median	Mini-mum	Maxi-mum	Sample size	Median	Mini-mum	Maxi-mum	Sample size	Median	Mini-mum	Maxi-mum	Sample size	Median	Mini-mum	Maxi-mum	Sample size	Median	Mini-mum	Maxi-mum	Sample size	Median	Mini-mum	Maxi-mum	Sample size	Median	Mini-mum	Maxi-mum	Trend
**Scenario GFDL-A2**																																					
**San Joaquin River**																																					
Mossdale	9	270.5	248	285	10	271.0	255	281	10	268.5	254	284	10	278.0	251	282	10	279.0	271	286	10	273.5	263	290	10	281.0	261	288	10	283.5	272	293	10	289.5	284	302	2.23**
Burns Cut	9	277.0	261	290	10	279.0	259	287	10	277.0	259	289	10	283.5	260	289	10	285.0	280	292	10	284.5	273	294	10	286.5	273	299	10	291.0	282	296	10	295.5	288	303	2.17***
Prisoners Point	9	260.5	224	280	10	256.5	236	278	10	251.5	234	265	10	263.0	248	280	10	270.5	254	281	10	269.5	260	286	10	275.5	258	287	10	280.5	262	287	10	284.0	274	301	3.68**
Jersey Point	9	264.0	217	278	10	264.0	251	285	10	260.5	240	279	10	268.5	251	281	10	277.5	258	286	10	276.0	265	291	10	281.5	261	288	10	288.0	278	295	10	290.5	287	303	3.79**
Antioch	9	261.0	217	282	10	257.0	236	279	10	251.5	234	277	10	263.5	248	280	10	275.0	254	282	10	270.5	261	288	10	278.5	258	288	10	281.5	266	290	10	286.0	279	302	3.91**
**Sacramento River**																																					
Hood	9	232.5	207	262	10	249.5	216	276	10	236.0	216	251	10	248.0	229	266	10	255.0	244	270	10	260.5	240	274	10	266.5	231	285	10	277.5	259	285	10	280.0	258	300	5.79***
Rio Vista	9	252.5	215	280	10	251.0	226	277	10	248.0	231	263	10	256.0	230	278	10	270.0	250	276	10	266.0	249	285	10	269.0	252	287	10	280.5	261	287	10	284.0	274	301	4.44**
Decker Island	9	246.5	215	270	10	253.0	235	281	10	251.5	235	265	10	254.5	248	277	10	276.0	255	283	10	271.5	261	288	10	278.5	258	288	10	283.5	268	293	10	287.5	281	302	5.44***
**North Delta**																																					
Upper Cache Slough	8	215.0	207	260	8	222.5	207	276	9	227.0	198	248	10	225.5	210	258	10	248.5	229	262	10	242.0	218	271	10	257.0	226	278	10	272.0	246	279	10	280.0	253	299	8.10***
Miners Slough	8	216.0	210	260	9	222.0	214	259	10	227.0	199	249	10	227.5	216	256	10	251.0	230	263	10	242.5	222	272	10	257.5	227	277	10	272.5	248	279	10	279.0	254	282	7.99***
Liberty Island	9	252.5	208	273	10	253.0	217	281	10	249.5	228	264	10	253.0	229	280	10	270.5	250	282	10	269.0	259	288	10	275.0	256	287	10	280.5	261	289	10	284.5	274	301	4.63**
Deepwater Ship Channel	8	221.0	212	261	10	241.0	215	276	10	230.5	214	250	10	231.0	224	266	10	252.0	241	269	10	250.0	239	274	10	260.0	228	279	10	273.5	249	285	10	282.0	255	299	6.99**
Lower Cache Slough	8	217.0	211	261	10	234.0	214	276	10	229.5	200	250	10	228.0	217	265	10	252.0	239	269	10	243.5	222	274	10	258.5	227	279	10	273.5	248	285	10	282.0	254	300	7.53**
**Confluence**																																					
Mallard Island	8	226.0	216	262	10	235.5	215	276	10	229.0	213	251	10	236.0	228	266	10	254.0	243	270	10	257.0	239	274	10	265.5	230	286	10	277.5	249	285	10	280.0	257	300	7.27***
**Suisun Bay**																																					
Martinez	4	211.5	198	236	4	210.5	187	258	8	224.0	197	227	9	226.0	206	255	9	240.0	215	262	10	231.0	211	270	10	254.0	226	278	10	268.5	247	282	10	273.5	253	281	8.12**
**Scenario GFDL-B1**																																					
**San Joaquin River**																																					
Mossdale	9	261.0	250	289	10	266.0	243	277	10	271.5	243	285	10	274.0	260	288	10	274.0	260	285	10	277.0	267	292	10	272.0	265	285	10	274.5	267	289	10	277.0	257	286	1.56**
Burns Cut	9	267.0	258	293	10	275.5	266	281	10	280.0	255	286	10	279.0	269	289	10	277.5	275	288	10	281.5	273	297	10	277.5	273	287	10	282.0	275	293	10	283.0	268	291	1.35*
Prisoners Point	9	252.5	229	272	10	250.5	236	274	10	255.0	241	270	10	260.5	232	277	10	260.5	247	274	10	265.0	241	277	10	267.5	251	273	10	267.0	258	284	10	261.0	252	272	1.88**
Jersey Point	9	253.5	231	288	10	261.5	242	275	10	259.0	236	277	10	265.0	256	284	10	269.5	251	284	10	271.0	242	292	10	274.0	257	285	10	274.0	265	289	10	270.5	259	279	2.36**
Antioch	9	252.0	228	276	10	250.5	223	274	10	255.0	241	274	10	262.0	232	278	10	260.0	241	275	10	266.0	241	288	10	269.0	252	275	10	268.0	259	286	10	263.0	252	273	2.14**
**Sacramento River**																																					
Hood	9	234.5	211	267	10	233.0	217	249	9	251.0	235	260	10	251.5	223	276	10	246.0	223	267	10	256.0	222	272	10	261.5	230	270	10	256.0	246	274	10	256.5	249	264	3.04**
Rio Vista	9	238.5	212	272	10	238.5	220	269	10	254.0	238	262	10	256.0	229	277	10	256.0	233	268	10	261.0	238	276	10	265.5	251	272	10	264.5	257	283	10	260.0	251	266	3.20**
Decker Island	9	246.5	215	276	10	247.0	221	274	9	256.0	242	274	10	259.0	232	280	10	257.5	241	277	10	263.5	239	289	10	271.0	254	277	10	268.0	261	285	10	265.0	253	274	2.86**
**North Delta**																																					
Upper Cache Slough	9	217.5	193	260	7	213.0	196	250	7	241.0	230	258	10	245.5	215	258	8	233.0	208	259	9	252.0	216	267	10	252.0	171	266	10	248.5	200	272	9	253.0	239	264	4.62**
Miners Slough	9	217.5	195	260	9	214.0	198	250	8	237.5	211	258	10	242.5	217	259	9	231.0	206	259	9	254.0	219	267	10	252.0	220	267	10	248.5	201	272	9	255.0	241	263	4.90**
Liberty Island	9	237.0	211	272	10	244.5	217	274	10	254.0	180	270	10	259.5	224	278	10	256.0	232	275	10	259.5	238	277	10	270.0	251	275	10	266.0	259	285	10	259.5	250	272	2.58*
Deepwater Ship Channel	9	224.5	198	262	9	215.0	198	251	8	250.5	214	259	10	250.0	218	275	10	238.5	222	267	10	254.0	220	268	10	261.5	229	272	10	250.5	224	273	10	255.0	246	264	4.24*
Lower Cache Slough	9	224.5	195	261	9	214.0	198	251	8	245.0	213	259	10	249.5	217	275	10	235.0	207	267	9	254.0	242	268	10	261.0	220	272	10	250.0	223	273	10	254.0	246	264	4.38*
**Confluence**																																					
Mallard Island	9	233.5	197	262	9	215.0	199	248	8	251.0	213	260	10	250.0	221	275	10	244.5	210	267	10	255.0	220	272	10	261.0	229	270	10	251.0	225	274	10	256.0	247	264	3.72*
**Suisun Bay**																																					
Martinez	2	227.0	217	249	5	213.5	195	245	6	220.0	209	248	9	243.0	215	251	7	220.0	192	257	8	250.0	206	265	8	255.0	221	265	9	244.0	198	270	9	241.0	214	257	NS
**Scenario PCM-A2**																																					
**San Joaquin River**																																					
Mossdale	9	268.5	245	282	10	262.5	252	277	10	276.0	262	285	10	273.0	254	294	10	276.5	253	289	10	270.0	263	287	10	277.0	267	289	10	280.0	266	295	10	287.5	264	308	2.13**
Burns Cut	9	273.5	257	285	10	275.0	258	289	10	279.5	270	290	10	280.0	258	298	10	282.0	270	295	10	283.0	271	293	10	282.0	273	294	10	287.5	276	300	10	292.0	275	310	1.99***
Prisoners Point	9	240.5	223	275	10	254.0	237	270	10	254.5	231	271	10	260.5	234	283	10	253.5	243	285	10	262.0	251	276	10	269.0	252	285	10	276.5	259	289	10	277.0	264	294	4.10**
Jersey Point	9	243.0	223	277	10	255.5	243	273	10	255.0	232	274	10	269.5	252	292	10	260.0	246	291	10	268.0	259	287	10	274.5	255	297	10	277.0	268	291	10	288.0	268	308	4.70**
Antioch	9	240.5	207	276	10	252.5	236	271	10	255.0	230	272	10	265.0	234	289	10	253.5	243	288	10	262.0	251	277	10	270.5	252	287	10	278.5	260	293	10	279.5	265	297	4.37**
**Sacramento River**																																					
Hood	9	221.0	203	256	9	237.5	225	259	10	230.5	223	257	10	243.0	223	264	10	249.5	227	283	10	239.5	215	270	10	257.5	237	271	10	253.5	242	277	10	268.0	253	291	4.78**
Rio Vista	9	233.0	206	275	10	250.0	231	269	10	239.0	225	270	10	259.5	230	282	10	252.0	235	285	10	256.5	239	276	10	266.5	245	284	10	273.0	255	290	10	274.0	264	294	4.75**
Decker Island	8	235.0	205	276	10	251.5	236	271	10	241.0	229	270	10	262.0	237	283	10	253.5	242	289	10	265.0	251	277	10	271.0	247	295	10	275.0	261	290	10	282.5	265	299	5.39***
**North Delta**																																					
Upper Cache Slough	3	208.0	196	221	9	220.0	172	258	7	220.5	203	246	10	224.5	202	261	10	224.0	206	281	9	233.5	224	258	10	241.0	221	269	10	237.5	226	273	10	252.0	230	288	4.64***
Miners Slough	4	196.0	196	223	10	221.0	173	258	8	221.0	211	246	10	225.5	203	262	10	225.0	216	281	9	232.5	201	257	10	245.5	223	270	10	235.0	226	254	10	253.5	244	288	5.47***
Liberty Island	9	233.0	199	275	10	248.0	230	270	10	238.5	225	271	10	260.5	229	288	10	251.5	228	287	10	264.5	238	276	10	268.0	245	292	10	274.0	258	290	10	277.0	263	296	5.26***
Deepwater Ship Channel	5	212.5	199	241	10	228.5	174	259	10	224.0	222	256	10	229.0	218	264	10	242.5	198	283	10	245.0	229	262	10	256.5	232	275	10	247.5	231	274	10	262.5	251	289	5.63***

Lower Cache Slough	4	204.0	196	223	10	226.0	173	259	9	223.0	221	254	10	226.5	208	263	10	238.0	196	283	10	236.5	228	261	10	256.5	232	275	10	241.0	227	274	10	260.5	251	291	5.80**
**Confluence**																																					
Mallard Island	6	220.5	196	254	9	226.0	173	259	10	227.0	222	256	10	227.0	218	264	10	248.0	225	283	9	237.0	230	268	10	256.5	235	270	10	249.0	235	277	10	267.5	252	292	5.43***
**Suisun Bay**																																					
Martinez	1	220.0	220	220	2	219.5	216	223	0	NV	NV	NV	4	229.0	210	260	7	221.0	201	233	8	226.0	215	249	9	224.0	214	263	10	225.5	217	267	10	248.5	225	287	NS
**Scenario PCM-B1**																																					
**San Joaquin River**																																					
Mossdale	9	268.0	254	300	10	275.5	256	285	10	266.0	254	293	10	269.5	246	287	10	273.0	255	287	10	272.5	256	289	10	270.0	259	291	10	274.0	255	288	10	272.0	257	289	NS
Burns Cut	9	275.0	259	302	10	280.5	265	287	10	278.0	263	297	10	277.5	264	290	10	276.5	261	293	10	280.0	265	290	10	281.5	270	295	10	288.5	262	296	10	278.5	268	292	NS
Prisoners Point	9	247.5	205	269	10	251.0	227	274	10	253.5	237	270	10	253.5	242	277	10	260.0	231	275	10	255.5	231	283	10	259.5	244	289	10	262.0	237	286	10	258.0	241	275	1.48**
Jersey Point	9	255.0	204	275	10	259.0	236	277	10	259.5	252	292	10	254.0	245	283	10	265.5	235	286	10	265.5	234	287	10	266.5	249	292	10	270.5	239	289	10	263.5	256	278	1.56*
Antioch	9	246.0	200	269	10	251.5	227	275	10	256.0	236	291	10	253.5	242	278	10	263.0	230	279	10	256.5	231	284	10	261.5	244	291	10	263.0	232	287	10	259.5	241	276	1.71*
**Sacramento River**																																					
Hood	8	223.0	210	235	10	223.0	212	249	10	230.0	216	267	10	230.0	202	264	10	236.0	223	271	10	246.5	227	280	10	255.5	228	284	10	253.5	221	276	10	242.5	224	274	3.95**
Rio Vista	9	233.5	196	250	10	248.5	225	273	10	246.0	223	269	10	243.5	229	276	10	246.5	226	275	10	252.5	229	283	10	257.0	232	289	10	262.0	226	286	10	255.5	240	275	2.66**
Decker Island	8	236.0	217	252	10	247.5	222	273	10	250.0	224	277	10	247.0	230	276	10	256.5	230	277	10	256.0	231	284	10	258.5	243	292	10	264.5	230	288	10	257.0	242	277	2.68**
**North Delta**																																					
Upper Cache Slough	3	206.0	205	227	4	214.5	200	233	4	227.0	202	264	5	216.5	197	262	10	229.0	196	269	8	238.0	222	280	9	250.0	215	281	9	235.0	210	266	10	226.0	195	273	3.48*
Miners Slough	7	218.0	197	230	8	212.5	198	236	8	212.0	198	250	8	222.0	196	262	10	229.5	205	268	10	226.5	201	264	9	250.0	221	279	10	235.5	195	266	10	226.5	215	272	3.06*
Liberty Island	8	235.0	213	256	10	246.5	224	274	10	250.0	226	291	10	243.5	229	277	10	250.0	225	275	10	254.0	228	284	10	258.0	230	290	10	262.5	225	287	10	254.5	239	275	1.75*
Deepwater Ship Channel	7	221.5	199	234	9	219.0	211	237	9	222.0	204	265	9	226.5	200	263	10	232.0	208	270	10	232.5	223	280	10	251.5	226	282	10	252.0	216	274	10	233.0	217	274	3.5**
Lower Cache Slough	7	220.5	198	231	8	217.0	210	236	9	218.0	199	265	8	224.0	197	263	10	231.0	206	270	10	232.0	202	280	10	249.0	221	283	10	243.5	213	274	10	228.0	215	274	2.99*
**Confluence**																																					
Mallard Island	7	220.5	199	234	8	220.0	210	237	9	219.0	199	266	8	233.0	196	264	10	227.5	208	270	10	235.0	223	280	10	253.0	227	284	10	252.5	216	274	10	235.0	217	274	3.76*
**Suisun Bay**																																					
Martinez	2	205.0	199	211	2	228.5	226	231	1	224.0	224	224	1	238.0	216	260	4	219.0	202	223	4	220.5	215	262	4	214.5	199	219	6	222.0	205	261	6	224.0	220	268	NS
