# Supplementary material for: Coupled Downscaled Climate Models and Ecophysiological Metrics Forecast Habitat Compression for an Endangered Estuarine Fish
Source: PLoS One. 2016 Jan 21;11(1):e0146724. doi: 10.1371/journal.pone.0146724 (PMC4721863; doi:10.1371/journal.pone.0146724)
Supplement: S6 Table — (PDF) [file pone.0146724.s011.pdf]

S6 Table. Median, minimum, and maximum values for the number of days per year for the duration of the maturation window (last day of 24°C to beginning of the spawning window) during each decade from 2010-2099, for the adult life stage of Delta Smelt for the least-warming (PCM-B1), most-warming (GFDL-A2) and two intermediate (PCM-A2 and GFDL-B1) climate change scenarios. The significance value for Trend is from the Mann-Kendal test (NS, P≥0.05; *, P<0.05; **, P<0.01; ***, P<0.001; NA, no non-zero values; NT, fewer than 3 values so trend not calculated) and the number is the slope of a regression of decadal medians.

	2010-2019				2020-2029				2030-2039				2040-2049				2050-2059				2060-2069				2070-2079				2080-2089				2090-2099				
	Sample size	Median	Mini-mum	Maxi-mum	Sample size	Median	Mini-mum	Maxi-mum	Sample size	Median	Mini-mum	Maxi-mum	Sample size	Median	Mini-mum	Maxi-mum	Sample size	Median	Mini-mum	Maxi-mum	Sample size	Median	Mini-mum	Maxi-mum	Sample size	Median	Mini-mum	Maxi-mum	Sample size	Median	Mini-mum	Maxi-mum	Sample size	Median	Mini-mum	Maxi-mum	Trend
**Scenario GFDL-A2**																																					
**San Joaquin River**																																					
Mossdale	9	175.0	145	195	10	171.5	147	199	10	179.5	160	193	10	176.5	137	191	10	156.5	144	181	10	160.5	143	180	10	149.5	140	180	10	133.5	110	160	10	136.0	120	145	-5.77**
Burns Cut	9	176.0	160	190	10	170.0	160	195	10	176.5	161	191	10	170.5	152	191	10	154.5	147	173	10	159.0	128	177	10	148.0	142	169	10	141.5	119	169	10	136.5	122	161	-5.20**
Prisoners Point	9	188.0	174	223	10	185.5	166	208	10	198.5	192	220	10	184.0	166	209	10	170.5	156	188	10	170.5	155	184	10	158.5	147	184	10	149.5	125	162	10	142.5	138	167	-6.39***
Jersey Point	9	193.0	176	259	10	195.5	180	207	10	198.5	174	220	10	191.0	167	214	10	173.0	155	199	10	170.5	124	192	10	153.5	141	186	10	148.5	126	169	10	144.0	135	169	-7.46**
Antioch	9	194.0	173	232	10	189.0	168	209	10	203.5	175	221	10	186.5	168	211	10	165.5	154	188	10	172.0	153	185	10	155.0	146	191	10	148.5	122	169	10	143.5	124	175	-7.25**
**Sacramento River**																																					
Hood	9	237.0	198	260	10	222.0	192	250	10	229.5	210	256	10	215.0	196	246	10	202.5	183	222	10	197.0	168	224	10	183.5	152	222	10	169.5	158	196	10	160.0	145	186	-9.59***
Rio Vista	9	225.0	185	237	10	209.0	188	237	10	219.0	205	232	10	207.5	190	228	10	190.5	169	218	10	188.5	160	206	10	177.0	150	204	10	161.5	152	195	10	157.5	140	179	-8.59***
Decker Island	9	216.0	187	263	10	209.5	185	229	10	213.5	201	239	10	208.5	187	226	10	184.5	160	205	10	184.5	130	208	10	162.5	154	189	10	162.0	128	182	10	155.0	139	171	-8.54***
**North Delta**																																					
Upper Cache Slough	8	239.0	201	269	8	227.5	194	253	9	236.0	210	260	10	229.0	197	266	10	199.5	178	234	10	199.0	167	240	10	175.0	153	200	10	165.5	132	193	10	161.5	134	180	-10.8***
Miners Slough	8	240.0	202	266	9	230.0	195	256	10	232.0	210	253	10	226.5	198	265	10	205.0	178	233	10	196.0	169	236	10	181.0	156	209	10	171.0	133	193	10	163.0	142	180	-10.29***
Liberty Island	9	215.0	178	263	10	213.5	189	233	10	212.5	201	233	10	200.5	191	233	10	189.0	160	206	10	181.0	127	210	10	163.0	146	191	10	159.5	131	188	10	152.5	141	176	-9.48***
Deepwater Ship Chann	8	225.5	200	264	10	218.5	193	231	10	221.0	205	249	10	219.5	189	250	10	195.0	174	229	10	188.5	165	216	10	172.5	151	198	10	153.0	128	179	10	159.0	127	179	-9.84**
Lower Cache Slough	8	237.0	201	265	10	224.0	193	249	10	226.0	205	251	10	226.0	190	264	10	195.5	174	232	10	194.0	166	235	10	173.5	151	200	10	153.5	128	191	10	159.5	126	180	-10.98**
**Confluence**																																					
Mallard Island	8	231.5	195	261	10	221.5	191	252	10	231.5	210	255	10	224.0	196	247	10	198.5	171	222	10	189.5	160	217	10	180.5	150	221	10	165.5	147	200	10	157.5	142	180	-10.01***
**Suisun Bay**																																					
Martinez	4	257.0	223	267	4	253.0	200	281	8	246.5	233	271	9	238.0	203	257	9	208.0	180	253	10	218.5	174	241	10	190.0	175	226	10	174.0	159	203	10	172.0	144	190	-11.83***
**Scenario GFDL-B1**																																					
**San Joaquin River**																																					
Mossdale	9	195.0	169	206	10	178.5	166	221	10	166.5	127	217	10	164.0	155	182	10	165.5	143	187	10	164.5	126	185	10	157.5	133	198	10	160.5	141	193	10	155.0	133	176	-3.68**
Burns Cut	9	188.0	165	197	10	180.0	170	202	10	169.5	149	205	10	164.5	154	179	10	168.5	146	184	10	157.5	148	173	10	172.5	143	187	10	158.0	147	183	10	161.0	141	181	-2.92**
Prisoners Point	9	204.0	176	221	10	202.5	174	225	10	187.0	163	223	10	186.5	167	206	10	186.0	176	210	10	174.5	159	199	10	177.5	153	200	10	166.5	160	195	10	181.0	163	207	-3.85**
Jersey Point	9	215.0	181	220	10	201.5	183	227	10	194.0	156	218	10	185.5	173	204	10	185.0	162	225	10	183.5	151	212	10	179.5	156	209	10	175.0	145	198	10	183.0	158	200	-3.98***
Antioch	9	207.0	177	226	10	208.5	177	229	10	190.0	152	237	10	187.0	168	209	10	192.5	179	216	10	174.5	159	200	10	178.5	151	204	10	168.5	161	196	10	183.5	160	210	-4.16*
**Sacramento River**																																					
Hood	9	230.0	199	256	10	235.5	206	256	9	208.0	176	228	10	210.0	185	234	10	215.0	198	244	10	203.5	185	223	10	196.0	161	232	10	201.5	174	215	10	201.5	184	217	-4.11*
Rio Vista	9	230.0	194	252	10	228.5	186	253	10	205.5	174	243	10	205.5	184	227	10	214.0	192	234	10	194.0	183	208	10	188.0	160	208	10	187.5	169	204	10	198.5	180	216	-4.93**
Decker Island	9	225.0	195	259	10	224.5	187	249	9	204.0	167	222	10	206.5	178	241	10	207.0	174	232	10	191.5	173	224	10	188.5	164	214	10	189.0	164	205	10	194.5	179	209	-4.58*
**North Delta**																																					
Upper Cache Slough	9	251.0	209	279	7	245.0	193	275	7	203.0	147	232	10	204.5	185	248	8	225.0	182	247	9	200.0	174	235	10	208.0	162	273	10	202.0	151	232	9	197.0	156	209	-5.66*
Miners Slough	9	250.0	210	277	9	244.0	212	277	8	216.0	172	240	10	219.0	186	246	9	233.0	183	245	9	202.0	175	232	10	205.5	162	230	10	202.5	167	231	9	199.0	185	212	-6.11**
Liberty Island	9	228.0	198	262	10	221.0	185	252	10	206.0	167	279	10	193.5	179	240	10	207.0	167	237	10	188.0	166	222	10	185.5	157	216	10	187.5	147	207	10	197.0	179	213	-3.98*
Deepwater Ship Chann	9	237.0	207	273	9	242.0	193	272	8	202.5	147	235	10	197.5	180	244	10	208.0	180	238	10	198.5	162	234	10	186.0	154	220	10	192.5	151	228	10	195.0	178	205	-5.81*
Lower Cache Slough	9	237.0	208	277	9	244.0	210	272	8	205.0	147	236	10	201.5	181	246	10	217.5	180	245	9	198.0	162	211	10	186.5	154	229	10	193.0	151	230	10	195.5	179	207	-5.99**
**Confluence**																																					
Mallard Island	9	233.0	204	265	9	257.0	206	267	8	206.5	175	244	10	203.5	185	233	10	215.5	198	253	10	192.0	185	224	10	190.0	160	232	10	200.0	166	236	10	200.5	182	217	-5.76*
**Suisun Bay**																																					
Martinez	2	244.0	232	256	5	248.0	210	275	6	38.5	204	243	9	219.0	201	247	7	240.0	203	272	8	201.0	188	255	8	197.0	176	235	9	208.0	171	256	9	221.0	186	249	NS
**Scenario PCM-A2**																																					
**San Joaquin River**																																					
Mossdale	9	183.0	166	221	10	182.5	166	204	10	175.0	158	181	10	177.5	142	196	10	159.0	133	168	10	161.5	142	184	10	165.0	149	181	10	155.0	120	167	10	147.0	119	179	-4.38**
Burns Cut	9	180.0	176	211	10	176.5	162	193	10	174.0	157	189	10	171.0	142	194	10	162.0	146	180	10	160.5	142	182	10	163.0	148	177	10	153.5	131	169	10	147.5	140	176	-3.86***
Prisoners Point	9	223.0	169	234	10	194.0	181	210	10	198.0	185	218	10	191.5	176	218	10	187.0	157	200	10	180.5	160	192	10	180.0	156	198	10	164.5	140	190	10	159.5	149	184	-6.49***
Jersey Point	9	221.0	188	259	10	200.0	182	211	10	197.0	181	222	10	191.0	165	221	10	187.5	149	220	10	179.0	158	200	10	177.0	157	203	10	172.5	138	182	10	161.5	148	186	-6.21***
Antioch	9	229.0	187	251	10	194.5	183	211	10	198.5	186	221	10	201.0	151	219	10	187.0	154	201	10	180.5	161	200	10	182.0	155	198	10	164.0	135	191	10	155.0	149	187	-7.35**
**Sacramento River**																																					
Hood	9	253.0	212	276	9	214.0	196	245	10	223.0	207	240	10	224.5	193	255	10	207.5	169	230	10	206.5	173	244	10	205.0	182	237	10	200.5	172	209	10	192.5	167	205	-5.61**
Rio Vista	9	238.0	201	259	10	213.0	191	239	10	211.5	195	235	10	214.5	185	249	10	202.5	166	219	10	192.5	166	219	10	194.0	162	210	10	185.0	166	200	10	180.0	163	202	-6.22**
Decker Island	8	238.5	199	280	10	212.0	186	235	10	218.0	195	226	10	198.0	174	248	10	203.0	153	227	10	187.0	168	210	10	185.0	162	226	10	181.5	148	190	10	172.5	154	190	-7.21***
**North Delta**																																					
Upper Cache Slough	3	250.0	229	276	9	229.0	191	292	7	235.0	206	255	10	238.0	193	266	10	219.5	158	265	9	215.0	171	231	10	201.5	178	238	10	209.5	169	230	10	190.0	162	223	-6.48**
Miners Slough	4	262.5	231	277	10	235.5	191	292	8	234.0	206	247	10	233.5	192	265	10	219.5	159	247	9	218.0	187	243	10	206.0	178	237	10	209.0	169	228	10	195.0	165	219	-7.02***
Liberty Island	9	238.0	198	279	10	214.5	184	223	10	215.0	189	228	10	197.0	169	255	10	201.5	153	239	10	187.5	167	220	10	184.0	163	226	10	181.5	140	191	10	177.0	154	192	-6.37***

Deepwater Ship Chann	5	233.0	228	273	10	223.5	184	289	10	227.5	201	235	10	230.5	190	254	10	197.5	156	237	10	201.5	165	255	10	188.5	169	229	10	194.5	162	217	10	176.0	161	198	-7.03**
Lower Cache Slough	4	258.5	229	276	10	225.5	184	291	9	230.0	203	237	10	232.5	191	265	10	205.5	157	239	10	212.5	166	258	10	192.5	175	236	10	205.5	163	227	10	188.5	160	199	-7.25**
**Confluence**																																					
Mallard Island	6	249.0	215	282	9	232.0	202	275	10	226.5	207	241	10	233.5	191	256	10	211.5	168	227	9	209.0	172	223	10	201.5	182	238	10	202.0	147	226	10	192.0	165	216	-6.54**
**Suisun Bay**																																					
Martinez	1	237.0	237	237	2	242.0	239	245	0	NV	NV	NV	4	217.0	196	265	7	236.0	217	256	8	221.5	195	232	9	231.0	191	258	10	222.0	178	238	10	211.5	172	228	NS
**Scenario PCM-B1**																																					
**San Joaquin River**																																					
Mossdale	9	183.0	145	197	10	178.5	167	194	10	180.5	150	196	10	183.0	156	202	10	176.0	151	188	10	174.5	161	208	10	165.0	141	189	10	169.0	149	194	10	171.5	145	185	-1.90*
Burns Cut	9	177.0	147	192	10	177.0	159	190	10	175.5	155	191	10	177.0	155	195	10	174.0	149	181	10	169.5	165	204	10	163.0	142	182	10	157.0	147	185	10	168.0	151	175	-2.14**
Prisoners Point	9	202.0	185	243	10	208.5	188	243	10	194.0	173	213	10	196.0	184	208	10	185.5	176	225	10	194.5	177	219	10	186.0	156	203	10	181.0	160	208	10	185.5	159	200	-2.77*
Jersey Point	9	211.0	178	262	10	206.5	182	226	10	192.5	165	218	10	198.5	166	232	10	189.5	179	233	10	197.0	165	223	10	177.0	163	209	10	175.5	153	215	10	189.0	165	197	-3.56**
Antioch	9	203.0	188	255	10	212.0	194	245	10	188.0	168	228	10	198.0	185	215	10	186.0	162	230	10	198.5	178	222	10	180.0	155	205	10	183.5	157	211	10	185.5	159	203	-2.85*
**Sacramento River**																																					
Hood	8	245.0	231	268	10	241.0	211	258	10	243.5	189	258	10	232.5	192	276	10	216.5	185	245	10	218.5	184	237	10	207.0	168	235	10	209.0	180	234	10	197.5	171	243	-6.22**
Rio Vista	9	234.0	202	267	10	225.5	206	255	10	207.0	187	248	10	220.0	190	237	10	209.0	184	243	10	211.0	181	230	10	201.0	163	227	10	204.5	166	227	10	189.5	167	217	-4.37**
Decker Island	8	235.5	218	243	10	233.5	203	261	10	214.5	179	246	10	213.5	174	252	10	208.5	184	244	10	206.0	172	235	10	205.5	167	218	10	193.0	175	229	10	200.0	168	218	-4.82***
**North Delta**																																					
Upper Cache Slough	3	264.0	236	276	4	243.0	226	264	4	230.5	189	265	5	245.0	178	257	10	233.5	183	272	8	220.5	170	231	9	213.0	167	242	9	216.0	178	251	10	220.0	169	265	-5.28**
Miners Slough	7	249.0	230	276	8	249.5	226	272	8	244.5	208	265	8	240.0	178	269	10	232.5	185	263	10	231.5	198	262	9	213.0	176	243	10	216.5	179	247	10	219.5	170	245	-4.81**
Liberty Island	8	223.5	218	268	10	219.5	190	255	10	215.5	154	233	10	215.0	178	245	10	208.5	170	232	10	208.0	173	246	10	201.5	153	221	10	195.0	157	220	10	192.5	180	227	-3.74**
Deepwater Ship Chann	7	235.0	228	264	9	239.0	222	264	9	226.0	188	262	9	219.0	177	265	10	228.5	182	260	10	222.0	170	240	10	201.5	166	231	10	197.0	175	232	10	213.5	168	243	-4.3**
Lower Cache Slough	7	244.0	228	276	8	244.0	223	268	9	239.0	188	264	8	235.5	177	268	10	230.5	182	262	10	223.5	170	261	10	208.5	165	237	10	208.0	176	236	10	218.5	168	245	-4.72***
**Confluence**																																					
Mallard Island	7	249.0	234	262	8	244.0	222	268	9	247.0	193	266	8	230.5	191	281	10	233.0	185	244	10	228.5	184	245	10	203.0	168	237	10	209.0	172	237	10	204.5	170	242	-6.22**
**Suisun Bay**																																					
Martinez	2	255.0	255	255	2	238.0	229	247	1	221.0	221	221	1	244.0	244	244	4	246.5	202	253	4	242.0	238	252	4	243.5	204	266	6	238.5	199	250	6	223.5	177	246	NS
