# Supplementary material for: Coupled Downscaled Climate Models and Ecophysiological Metrics Forecast Habitat Compression for an Endangered Estuarine Fish
Source: PLoS One. 2016 Jan 21;11(1):e0146724. doi: 10.1371/journal.pone.0146724 (PMC4721863; doi:10.1371/journal.pone.0146724)
Supplement: S1 Table — (PDF) [file pone.0146724.s006.pdf]

S1 Table. Median, minimum, and maximum values for the number of days each year when mean daily water temperature is $\geq CLT_{max}$, during each decade from 2010-2099, for the juvenile life stage of Delta Smelt (June-December) for the least-warming (PCM-B1), most-warming (GFDL-A2) and two intermediate (PCM-A2 and GFDL-B1) climate change scenarios. The significance value for Trend is from the Mann-Kendal test (NS, $P \geq 0.05$; *, $P<0.05$; **, $P<0.01$; ***, $P<0.001$; NA, no non-zero values; NT, fewer than 3 values so trend not calculated) and the number is the slope of a regression of decadal medians.

	2010-2019			2020-2029			2030-2039			2040-2049			2050-2059			2060-2069			2070-2079			2080-2089			2090-2099			
	Median	Minimum	Maximum	Median	Minimum	Maximum	Median	Minimum	Maximum	Median	Minimum	Maximum	Median	Minimum	Maximum	Median	Minimum	Maximum	Median	Minimum	Maximum	Median	Minimum	Maximum	Median	Minimum	Maximum	Trend
**Scenario GFDL-A2**																												
**San Joaquin River**																												
Mossdale	28	11	47	41.5	19	79	41.5	26	71	45.5	25	69	59	39	86	71	56	111	82.5	62	110	102	72	128	117	94	130	10.75***
Burns Cut	59.5	24	89	61.5	58	100	71	46	89	67.5	51	111	94.5	62	120	99.5	77	120	102.5	94	135	125	94	133	128	105	140	9.33***
Prisoners Point	0	0	7	0	0	7	4.5	0	12	7.5	0	14	4	0	20	13	7	44	22.5	6	60	31	11	80	56.5	36	73	6***
Jersey Point	0	0	5	0	0	16	0	0	12	12	0	19	11	0	37	21	0	55	29	9	72	54.5	25	83	91.5	73	109	9.94***
Antioch	0	0	11	0	0	21	7.5	0	14	17.5	0	22	11.5	0	45	24	15	54	33.5	12	80	56	25	101	89	68	111	9.71***
**Sacramento River**																												
Hood	0	0	0	0	0	0	0	0	5	0	0	10	0	0	13	6.5	0	42	19	0	62	24.5	3	84	52.5	45	74	5.47**
Rio Vista	0	0	3	0	0	3	0	0	7	4	0	12	2	0	18	14.5	6	50	26	4	69	34	12	93	68	53	95	7.28***
Decker Island	0	0	0	0	0	0	0	0	5	0	0	9	2	0	13	4	0	43	13	0	68	34	2	74	73	54	101	7.07***
**North Delta**																												
Upper Cache Slough	0	0	0	0	0	0	0	0	0	0	0	0	0	0	2	0	0	2	2	0	16	5.5	0	38	26.5	2	35	2.11**
Miners Slough	0	0	0	0	0	0	0	0	0	0	0	0	0	0	2	0	0	4	2	0	20	6.5	0	41	31	14	37	2.46**
Liberty Island	0.5	0	10	0	0	12	5	0	15	12.5	0	18	16	1	32	19	1	51	27	12	73	42.5	16	79	72.5	55	100	9.51***
Deepwater Ship Channel	0	0	3	0	0	2	0	0	5	1	0	10	0	0	14	7	0	40	15	0	53	24	4	57	50.5	40	73	5.17**
Lower Cache Slough	0	0	0	0	0	0	0	0	0	0	0	0	0	0	3	0	0	9	3	0	26	12	0	43	34	18	43	2.97**
**Confluence**																												
Mallard Island	0	0	0	0	0	0	0	0	1	0	0	2	0	0	8	0	0	22	9.5	0	44	14.5	0	56	39	25	56	3.64***
**Suisun Bay**																												
Martinez	0	0	0	0	0	0	0	0	0	0	0	0	0	0	0	0	0	0	2.5	0	16	6.5	0	41	22.5	1	33	1.91**
**Scenario GFDL-B1**																												
**San Joaquin River**																												
Mossdale	31	13	65	28	5	41	43.5	4	80	42	30	76	50	18	77	51	22	75	61	43	111	48.5	26	81	71	51	94	4.43**
Burns Cut	55	37	79	59	42	74	68	30	88	69.5	56	101	72.5	40	92	82.5	57	103	97.5	64	115	81.5	69	107	92	58	109	4.79**
Prisoners Point	0	0	9	0	0	6	1.5	0	23	1	0	12	3	0	15	4.5	0	31	2.5	0	44	2	0	16	10	0	36	0.86*
Jersey Point	0	0	12	0	0	4	0	0	37	0	0	18	0	0	16	7.5	0	46	0	0	14	2	0	77	15	0	57	NS
Antioch	0	0	11	0	0	17	3	0	40	1.5	0	32	5.5	0	19	6.5	0	50	5.5	0	80	7	0	21	19	0	56	1.79**
**Sacramento River**																												
Hood	0	0	0	0	0	0	0	0	14	0	0	10	0	0	11	0	0	30	0	0	31	0	0	10	0	0	27	NA
Rio Vista	0	0	3	0	0	4	0	0	24	0	0	13	0	0	15	1	0	33	0	0	48	0	0	12	12	0	37	NS
Decker Island	0	0	0	0	0	0	0	0	20	0	0	10	0	0	6	0	0	29	0	0	0	0	0	51	0	0	36	NA
**North Delta**																												
Upper Cache Slough	0	0	0	0	0	0	0	0	3	0	0	3	0	0	7	0	0	0	0	0	0	0	0	5	0	0	7	NA
Miners Slough	0	0	0	0	0	0	0	0	3	0	0	3	0	0	5	0	0	0	0	0	0	0	0	4	0	0	9	NA
Liberty Island	0	0	14	0	0	10	4.5	0	27	3	0	23	3	0	17	11	0	34	3.5	0	17	7	0	67	16	0	45	2.04**
Deepwater Ship Channel	0	0	7	0	0	4	0	0	17	0	0	12	0	0	13	0	0	25	0	0	0	0	0	19	5	0	24	NT
Lower Cache Slough	0	0	0	0	0	0	0	0	5	0	0	5	0	0	8	0	0	2	0	0	0	0	0	6	0	0	13	NA
**Confluence**																												
Mallard Island	0	0	0	0	0	0	0	0	9	0	0	8	0	0	9	0	0	9	0	0	10	0	0	6	0	0	17	NA
**Suisun Bay**																												
Martinez	0	0	0	0	0	0	0	0	2	0	0	3	0	0	5	0	0	0	0	0	1	0	0	0	0	0	7	NA
**Scenario PCM-A2**																												
**San Joaquin River**																												

Mossdale	11.5	3	36	30	20	41	40	17	51	41.5	29	60	48	11	73	--	49	38	76	66.5	47	96	67	57	87	88	69	116	7.96***
Burns Cut	46.5	19	66	66.5	44	75	56.5	39	90	78.5	55	88	71	55	112	--	76	60	100	90	71	113	94.5	87	106	103	93	136	6.24**
Prisoners Point	0	0	0	0	0	4	0	0	0	0	0	6	0	0	11	--	0	0	5	3.5	0	20	13	4	24	16	0	31	1.83**
Jersey Point	0	0	0	0	0	3	0	0	0	0	0	4	0	0	24	--	0	0	7	13	0	43	26	6	42	39	2	47	4.33**
Antioch	0	0	4	0	0	6	0	0	0	0.5	0	8	1	0	16	--	1	0	32	15	0	43	27	12	42	38	7	48	4.39***
**Sacramento River**																													
Hood	0	0	0	0	0	0	0	0	0	0	0	0	0	0	5	--	0	0	0	0	0	9	2	0	12	6	0	26	NT
Rio Vista	0	0	0	0	0	0	0	0	0	0	0	3	0	0	10	--	0	0	2	0	0	26	10	2	25	17	0	37	NT
Decker Island	0	0	0	0	0	0	0	0	0	0	0	0	0	0	3	--	0	0	0	0	0	28	5	0	15	13	0	35	NT
**North Delta**																													
Upper Cache Slough	0	0	0	0	0	0	0	0	0	0	0	0	0	0	0	--	0	0	0	0	0	0	0	0	0	0	0	9	NA
Miners Slough	0	0	0	0	0	0	0	0	0	0	0	0	0	0	0	--	0	0	0	0	0	0	0	0	0	0	0	10	NA
Liberty Island	0	0	3	0	0	5	0	0	0	2.5	0	7	1	0	17	--	2.5	0	9	11	0	31	20.5	9	29	29	6	42	3.33***
Deepwater Ship Channel	0	0	0	0	0	0	0	0	0	0	0	3	0	0	7	--	0	0	0	0	0	10	7.5	0	18	6	0	25	NA
Lower Cache Slough	0	0	0	0	0	0	0	0	0	0	0	0	0	0	0	--	0	0	0	0	0	0	0	0	0	0	0	10	NA
**Confluence**																													
Mallard Island	0	0	0	0	0	0	0	0	0	0	0	0	0	0	1	--	0	0	0	0	0	0	0	0	2	0	0	11	NA
**Suisun Bay**																													
Martinez	0	0	0	0	0	0	0	0	0	0	0	0	0	0	0	--	0	0	0	0	0	0	0	0	0	0	0	0	NA
**Scenario PCM-B1**																													
**San Joaquin River**																													
Mossdale	21.5	4	56	28	10	50	25	5	47	30	12	55	33	29	60		41	32	54	55	17	67	63	23	77	49	22	74	4.77**
Burns Cut	41.5	23	81	54	29	85	60	50	89	56	40	85	66.5	48	93		70	55	82	74	59	113	90.5	43	102	77	59	96	4.89***
Prisoners Point	0	0	8	0	0	0	0	0	11	0	0	4	0.5	0	3		0	0	0	0	0	4	0	0	16	0	0	16	NT
Jersey Point	0	0	0	0	0	3	0	0	12	0	0	0	0	0	9		0	0	0	0	0	1	0	0	23	0	0	20	NA
Antioch	0	0	16	0	0	6	0	0	17	0	0	7	0	0	12		0	0	1	0	0	7	0	0	30	0	0	23	NA
**Sacramento River**																													
Hood	0	0	0	0	0	0	0	0	0	0	0	0	0	0	0		0	0	0	0	0	0	0	0	11	0	0	7	NA
Rio Vista	0	0	5	0	0	0	0	0	5	0	0	0	0	0	0		0	0	0	0	0	0	0	0	15	0	0	13	NA
Decker Island	0	0	0	0	0	0	0	0	0	0	0	0	0	0	0		0	0	0	0	0	0	0	0	10	0	0	7	NA
**North Delta**																													
Upper Cache Slough	0	0	0	0	0	0	0	0	0	0	0	0	0	0	0		0	0	0	0	0	0	0	0	0	0	0	0	NA
Miners Slough	0	0	0	0	0	0	0	0	0	0	0	0	0	0	0		0	0	0	0	0	0	0	0	0	0	0	0	NA
Liberty Island	0	0	0	0	0	5	0	0	15	0	0	6	0	0	7		0	0	0	0	0	8	0	0	19	0	0	18	NA
Deepwater Ship Channel	0	0	0	0	0	0	0	0	0	0	0	0	0	0	0		0	0	0	0	0	0	0	0	13	0	0	12	NA
Lower Cache Slough	0	0	0	0	0	0	0	0	0	0	0	0	0	0	0		0	0	0	0	0	0	0	0	0	0	0	0	NA
**Confluence**																													
Mallard Island	0	0	0	0	0	0	0	0	0	0	0	0	0	0	0		0	0	0	0	0	0	0	0	0	0	0	0	NA
**Suisun Bay**																													
Martinez	0	0	0	0	0	0	0	0	0	0	0	0	0	0	0		0	0	0	0	0	0	0	0	0	0	0	0	NA
